# Grape-Pi: graph-based neural networks for enhanced protein identification in proteomics pipelines

**DOI:** 10.1093/bioadv/vbaf095

**Published:** 2025-04-26

**Authors:** Chunhui Gu, Seyyed Mahmood Ghasemi, Yining Cai, Johannes F Fahrmann, James P Long, Hiroyuki Katayama, Chong Wu, Jody Vykoukal, Jennifer B Dennison, Samir Hanash, Kim-Anh Do, Ehsan Irajizad

**Affiliations:** Department of Biostatistics, The University of Texas MD Anderson Cancer Center, Houston, TX 77030, United States; Department of Clinical Cancer Prevention, The University of Texas MD Anderson Cancer Center, Houston, TX 77030, United States; Department of Biostatistics, The University of Texas MD Anderson Cancer Center, Houston, TX 77030, United States; Department of Clinical Cancer Prevention, The University of Texas MD Anderson Cancer Center, Houston, TX 77030, United States; Department of Clinical Cancer Prevention, The University of Texas MD Anderson Cancer Center, Houston, TX 77030, United States; Department of Clinical Cancer Prevention, The University of Texas MD Anderson Cancer Center, Houston, TX 77030, United States; Department of Biostatistics, The University of Texas MD Anderson Cancer Center, Houston, TX 77030, United States; Department of Clinical Cancer Prevention, The University of Texas MD Anderson Cancer Center, Houston, TX 77030, United States; Department of Biostatistics, The University of Texas MD Anderson Cancer Center, Houston, TX 77030, United States; Department of Clinical Cancer Prevention, The University of Texas MD Anderson Cancer Center, Houston, TX 77030, United States; Department of Clinical Cancer Prevention, The University of Texas MD Anderson Cancer Center, Houston, TX 77030, United States; Department of Clinical Cancer Prevention, The University of Texas MD Anderson Cancer Center, Houston, TX 77030, United States; Department of Biostatistics, The University of Texas MD Anderson Cancer Center, Houston, TX 77030, United States; Department of Biostatistics, The University of Texas MD Anderson Cancer Center, Houston, TX 77030, United States; Department of Clinical Cancer Prevention, The University of Texas MD Anderson Cancer Center, Houston, TX 77030, United States

## Abstract

**Motivation:**

Protein identification via mass spectrometry (MS) is the primary method for untargeted protein detection. However, the identification process is challenging due to data complexity and the need to control false discovery rates (FDR) of protein identification. To address these challenges, we developed a graph neural network (GNN)-based model, Graph Neural Network using Protein–Protein Interaction for Enhancing Protein Identification (Grape-Pi), which is applicable to all proteomics pipelines. This model leverages protein–protein interaction (PPI) data and employs two types of message-passing layers to integrate evidence from both the target protein and its interactors, thereby improving identification accuracy.

**Results:**

Grape-Pi achieved significant improvements in area under receiver-operating characteristic curve (AUC) in differentiating present and absent proteins: 18% and 7% in two yeast samples and 9% in gastric samples over traditional methods in the test dataset. Additionally, proteins identified via Grape-Pi in gastric samples demonstrated a high correlation with mRNA data and identified gastric cancer proteins, like MAP4K4, missed by conventional methods.

**Availability and Implementation:**

Grape-Pi is freely available at https://zenodo.org/records/11310518 and https://github.com/FDUguchunhui/GrapePi.

## 1 Introduction

Mass spectrometry (MS) is a widely used high-throughput technique for detecting and analyzing proteins in biological samples (Chen and Yates 2007). It can identify several thousand proteins in a single run. The basic principle of shotgun proteomics is first to digest the proteins into peptides using enzymes (e.g. trypsin) and separate the peptides by reverse-phase chromatography. The separated peptides are fed into the mass spectrometer, in which they are ionized and broken down into smaller fragments (fragment ions). The resulting spectrum is matched to the theoretical peptide spectrum predicted for each peptide from a database of known protein sequences to identify the proteins in the sample (Nesvizhskii 2010). In shotgun proteomics, the identification is two-stage, from matching spectrum to peptides and then peptides to proteins.

In general, the number of identified proteins can be several thousand, depending on the size of the search protein sequence database and the tolerance of the search parameters (Cardoza *et al.* 2012). A critical challenge lies in accurately discerning proteins that are truly present (positive proteins) in the sample from those absent proteins (negative proteins). Usually, a protein identification score (a.k.a. protein score) is calculated for each identified protein based on evidence from their corresponding peptides. The protein score can be used to calculate protein probability and control the global false discovery rate (FDR) of identified positive proteins, typically using a target-decoy approach (Elias and Gygi 2010).

The above FDR approach relies on the discriminating power of protein score (discrepancy in the distributions of protein score between positive proteins and negative proteins). Controlling of FDR usually comes with the cost of fewer identified positive proteins [a.k.a. higher false negative rate (FNR)]. Sometimes, to further ensure the reliability of the findings, proteins without distinct peptide evidence or supported by a single peptide are removed (Nesvizhskii and Aebersold 2005, Li *et al.* 2009). Consequently, some true positive proteins are filtered due to physical properties of proteins (such as size and hydrophobicity) or low sensitivity for low-abundant proteins (the abundance-driven identification problem) during mass spectrometry (MS) (Nesvizhskii *et al.* 2003, Nesvizhskii 2010). This conservativeness prevents us from getting a holistic view of the protein picture in a sample and identifying potential biomarkers. A recent study showed that most biomarkers are predominantly among the low-abundant proteins, indicating the importance of the comprehensive identification of low-abundant proteins (Tognetti *et al.* 2022).

To mitigate this issue, researchers have employed various approaches, integrating the protein score or protein probability with additional information, such as predicted peptide detectability, external data (such as transcriptomics data), and interaction networks (Li *et al.* 2009, Ramakrishnan *et al.* 2009, Nusinow *et al.* 2012, Zhong *et al.* 2017). Incorporating the protein–protein interaction (PPI) network in shotgun proteomics is an established approach since it reflects the well-supported concept that proteins work together to perform their functionality (Jones and Thornton 1996, Hartwell *et al.* 1999).

Traditionally, the additional information was jointly modeled with protein scores. However, strong assumptions were often needed for such modeling (Casadio *et al.* 2022). Graph models have proven particularly useful for proteomics, where the inherent structure of protein data can be effectively represented as a graph with proteins severing as nodes and PPIs forming the connecting edges. Li *et al.* (2009) developed an approach known as the clique enrichment analysis within a protein network. This method involves the identification of cliques, defined as fully interconnected subgraphs where each protein is directly linked to every other protein within the clique. Non-confident proteins in a clique were assigned a label of present if the enrichment score of that clique surpassed a predefined threshold. This assumed that a non-confident protein was more likely to be present in a sample if it was interconnected with other confidentially identified proteins. Despite the utility of such a model simplifying the complexity of protein networks, it had limitations due to its exclusive reliance on one specific type of subgraph. Moreover, its inability to incorporate additional features of proteins or interactions also impedes it from exploiting the full potential of protein networks.

Recently, the emergence of graph neural networks (GNNs) as a deep learning architecture has introduced a novel and potent method for the examination of proteomics data in the context of the PPI network. A distinct advantage of GNNs over alternative deep learning architectures lies in their capability to assimilate information from the local neighborhood for nodes in the graph (Zhou *et al.* 2020), enabling GNNs to achieve state-of-art performance in key areas of proteomics research, including discovery of protein interactions (Jha *et al.* 2022), analysis of protein structure (Strokach *et al.* 2020), and predication of protein function (Gligorijević *et al.* 2021, Zuobai Zhang 2023). This aggregation of neighbor information could also be beneficial for greatly enhancing protein identification with a broad spectrum of information that cannot be achieved in earlier network models. However, to the best of our knowledge, no prior studies have utilized GNNs to improve protein identification in MS data via the PPI network.

In this study, we introduced a new framework called GNN using Protein–protein interaction for Enhancing Protein Identification (Grape-Pi) that leveraged semi-supervised GNNs to utilize PPIs data for enhanced protein identification in shotgun proteomics. Our exploration focused on two distinct GNN message-passing layers: GCNConv, representing the spectral convolutions, and SAGEConv, representing spatial convolutions (Wu *et al.* 2021). We used GraphGym, a versatile platform for evaluating design space, to determine the optimal hyperparameter set. Our findings demonstrated the universal applicability of this method in enhancing protein identification across a variety of MS platforms and biology samples with different complexities. Finally, we discussed potential challenges encountered during model training and the future directions for refining the model.

## 2 Methods

### 2.1 Shotgun proteomics data

#### 2.1.1 Dataset 1 and 2: yeast (rich medium)

Yeast is a microorganism whose proteome consists of ∼6700 proteins in UniProt (Swiss-Prot) and is often used to validate new MS methodology. Therefore, MS proteomics data were available publicly to create training datasets and the protein reference set, allowing evaluation of the performance of our Grape-Pi model with other existing models on the same data. The yeast (rich medium) data and protein reference set were sourced from Ramakrishnan *et al.* (2009), with sample preparation details in the original paper. All data used in this section can be found at: http://www.marcottelab.org/MSpresso/roc_and_scores/MAIN_PAPER/ypd_orbi_self-yp4gte2-or-notyp4_no-tm/. Cell lysate from wild-type yeast grown in the rich medium was analyzed on Thermo LTQ OrbiTrap (yeast-OrbiTrap) and the older Thermo DecaPlus LCQ (yeast-LCQ) mass spectrometers. PPI information was downloaded from the STRING v12.0. The PPIs from the STRING database had a confidence score; for the two yeast datasets, we used all PPIs without applying any filtering.

The protein reference set was generated from a pool of another four independent MS-based proteomics yeast (rich medium) datasets (Washburn *et al.* 2001, Peng *et al.* 2003, de Godoy *et al.* 2006, Chi *et al.* 2007). Proteins presented in at least two of the four datasets were treated as positive proteins (YP4gte2 rule), and proteins absent from all four datasets were treated as negative proteins. The yeast-OrbiTrap and yeast-LCQ protein datasets had 3208 proteins. Using this protein reference set for creating ground-truth labels, 1433 were labeled as positive proteins and 1776 were marked as negative proteins. The two datasets we used here only contained two variables. One is the protein probability output directly from the MS analysis software ProteinProphet without additional argumentation. We referred to it as “raw protein probability” (or “raw probability” for short) to distinguish it from the updated “predicted protein probability” (“predicted probability” for short) from the model. The second variable is the corresponding mRNA concentration measured under similar experimental conditions for each sample (Ramakrishnan *et al.* 2009).

#### 2.1.2 Dataset 3: Primary cell gastric cancer sample

The third dataset we considered was a more complex cancer cell sample. Samples are run through Waters SYNAPT G2-Si ion mobility-assisted DIA mode. The spectra are matched with Waters ProteinLynx Global SERVER™ (PLGS) and then searched using the combination of UniProtKB Swiss-Prot and TrEMBL databases (∼92 000 accessions). The PLGS workflow is set to three missed cleavages and without FDR filtering. Detailed information about the sample preparation can be found in Supplementary Biology Methods. Only Swiss-Prot proteins (∼20 000) were used in this study. Intact proteins in each sample were separated into 23 fractions using reverse-phase high-performance liquid chromatography (RP-HPLC) before being injected into liquid chromatography–mass spectrometry (LC-MS) for maximizing protein detection already. This dataset was used to evaluate how Grape-Pi performs in a sophisticated real-world dataset. PPI information was downloaded from the STRING database v12.0 (https://string-db.org/cgi/download? sessionId=b1wBXWoL3WRb&species_text=Saccharomyces+cerevisiae). Due to the huge number of human PPIs from the STRING database and more lower-end concentrated confidence score distribution compared with the yeast dataset (Supplementary Fig. S2), only PPIs with a confidence score of 400 or higher were used, corresponding to medium-level evidence. A sensitivity analysis was performed using different confidence thresholds to construct the interaction network. We observed slightly degenerated performance as more confident, but also fewer interactions were used in GCNConv, and the performance of SAGEConv is stable (Supplementary Fig. S6). This is consistent with the findings of Nusinow *et al.* (2012).

We utilized eight primary cell gastric cancer (PCGC) sample datasets from different patients. One dataset was used for training and validation, while the remaining seven were used for generating protein existence labels. The original Waters PLGS did not output raw probabilities. We assumed a mixture model protein score distribution among negative and positive proteins. Then, the expectation–maximization (EM) algorithm similar to Ma *et al.* (2012) was used for calculating raw probability (see Supplementary Methods for more details). The average raw probability across the seven samples was used as the ground-truth protein probability for each protein, and proteins with ground-truth probabilities >.7 were considered positive proteins and <.3 as negative proteins. The remaining proteins were unlabeled. This resulted in 4615 positive proteins and 7922 negative proteins. Following the data preparation, we developed a custom dataset class to import these data files into a format compatible with PyTorch Geometric, facilitating model tuning and downstream analysis (flowchart in Supplementary Figures). Detailed information about the structure of the dataset can be found in Supplementary Methods. Additionally, we tested the performance improvement using different labeling thresholds and observed a similar extent of improvement (Supplementary Fig. S5).

### 2.2 Experiment

**Figure 1.**
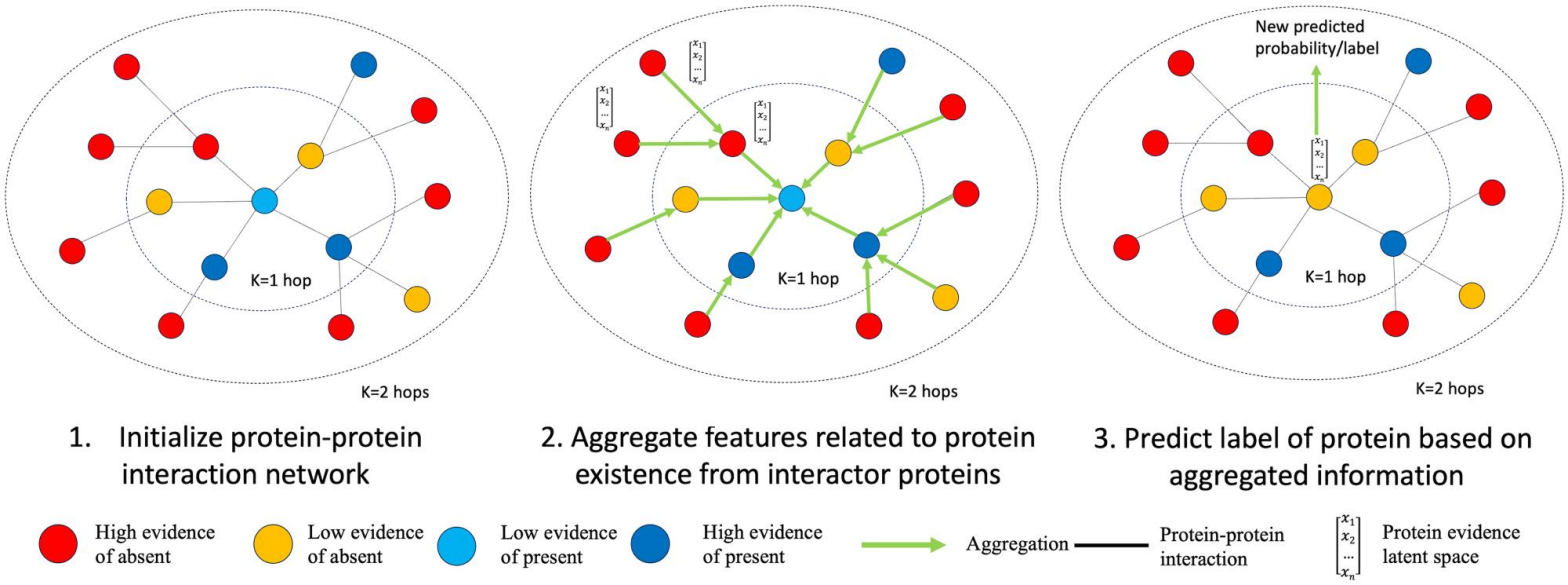
Illustration of protein evidence updating based on protein interactors by message-passing mechanism of graph neural network. Each node represents a protein, and the edge represents a known interaction between two proteins. Red dots, yellow dots, dark blue dots, and cyan dots represent proteins with high raw evidence of being present, low raw evidence of being present, high raw evidence of being absent, and low raw evidence of being absent, respectively. Left graph: the initial state of a target protein and its protein interactors in the protein–protein interaction network (PPIN). Middle graph: information in the form of embedding is aggregated from outer protein interactors (indirect interactors) into inner interactors (direct interactors) and finally into the target proteins. Right graph: the final embedding is used to predict the presence of the target protein. All proteins in the PPIN are updated as illustrated by the target protein either sequentially or spontaneously, depending on the type of the message-passing layer.

We assessed two models with different GNN message-passing layers (GCNConv and SAGEConv) and evaluated their performance in labeled proteins. Under the graph model setting, each node represents a protein; each edge represents a known interaction between two proteins. Information useful for deciding protein existence is aggregated from interactors based on the PPI network for each protein in the network, and the final embedding of proteins is fed into a classifier to decide protein existence (Fig. 1). The labeled data in each dataset was randomly split into 60/20/20 partitions for training, validation, and test datasets, and the sample size in each dataset can be found in Supplementary Table S2.

**Figure 2.**
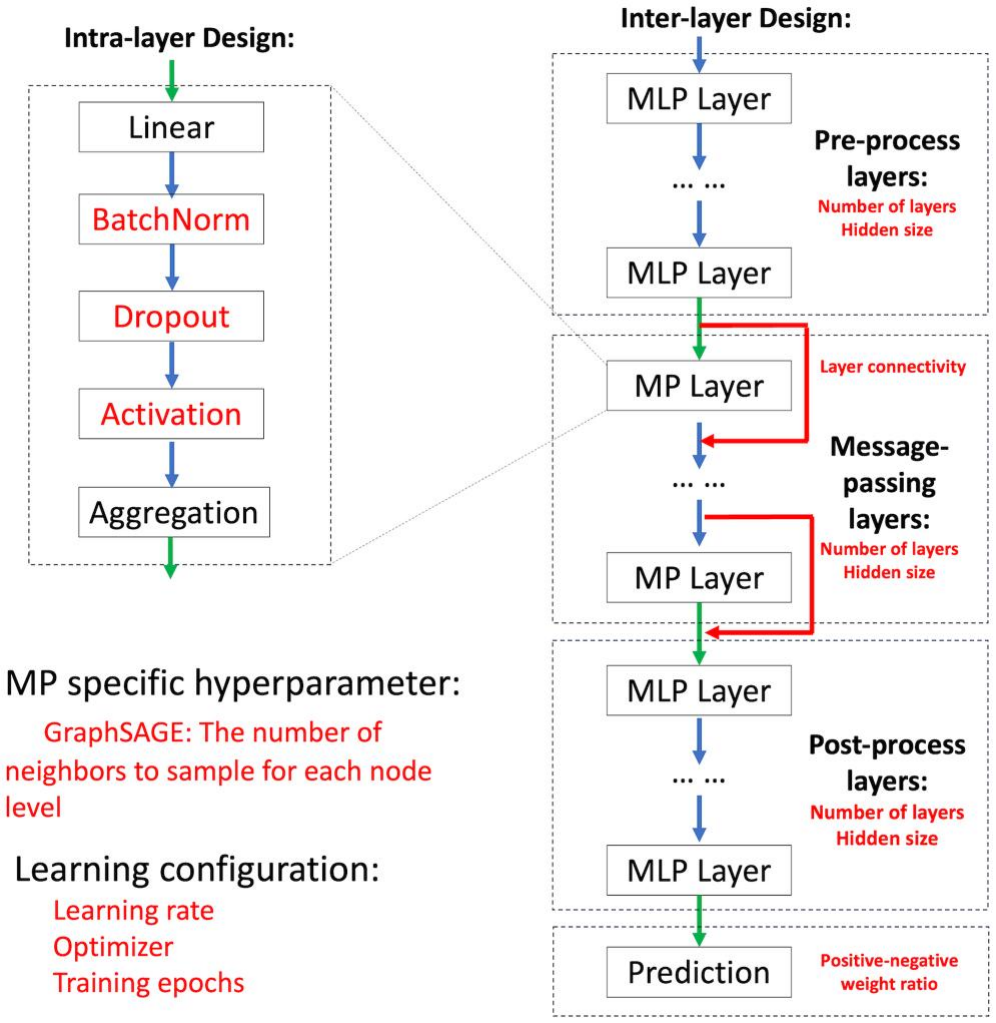
Model design and hyperparameter components. The framework follows a modular design with intralayer and interlayer structures. Intralayer design includes linear transformation, batch normalization, dropout, activation, and neighborhood aggregation. The interlayer design consists of three stages: preprocess, message-passing, and postprocess layers, each utilizing MLPs. The model hyperparameters include neighbor sampling (GraphSAGE), learning rate, optimizer, and training epochs, with a final prediction layer completing the architecture.

We evaluated a variety of hyperparameters, especially hyperparameters that are unique to GNN, such as the number of message-passing layers and the number of neighbor sizes in SAGEConv (Fig. 2, Supplementary Table S1) using the training dataset and picked the best model in terms of area under receiver-operating characteristic curve (ROC AUC) on the validation dataset. A detailed description of algorithms and hyperparameter tuning experiment setup for the two approaches is available in Supplementary Methods.

We illustrated Grape-Pi models, which were constructed with raw protein probability as the only node feature and PPIs as the unweighted edges. Based on design space exploration, the following choices were made for model design and hyperparameter. A single layer for preprocessing and post-processing steps for both SAGEConv and GCNConv were selected during the training processes. The optimal number of message-passing layers was one for both models, implying that the immediate interactor of a protein provided the most significant insight for predicting its presence. For the Grape-Pi-GCNConv model, a dropout rate of 0.3 achieved better performance on average for the regularization effect. In contrast, the Grape-Pi-SAGEConv model achieved better performance without using dropout. The SAGEConv aggregation operates by sampling a fixed number of neighbors during training and using all neighbors during the evaluation, inherently providing a regularization effect in preventing overfitting (Supplementary Fig. S3).

## 3 Results

### 3.1 Model evaluation on test dataset

To evaluate how accurate Grape-Pi model works for protein detection, we estimated the AUC performance of two proposed models (GCNConv and SAGEConv) as well as raw probability for predicting confidence protein. Our Grape-Pi models demonstrated a notable improvement for accurate protein detection in the yeast-LCQ dataset with respective AUC performance of 0.81 and 0.63 for Grape-Pi-GCNConv and raw probability. In the Yeast-ORBI dataset, Grape-Pi-GCNConv yielded an AUC performance of 0.91, while raw probability had an AUC of 0.82. In the gastric cancer samples, Grape-Pi showed a consistently high performance of 0.91 compared to the 0.82 performance of the raw probability (Table 1).

**Table 1.** Performance of Grape-Pi-SAGEConv and Grape-Pi-GCNConv with the best hyperparameter combination on three datasets.^a^

	ROC AUC		
Dataset	Raw probability	Grape-Pi-GCNConv	Grape-Pi-SAGEConv
Yeast-LCQ	0.63	0.81 (0.78–0.84)	0.76 (0.72–0.80)
Yeast-ORBI	0.84	0.91 (0.88–0.92)	0.90 (0.88–0.92)
PCGC	0.82	0.91 (0.90–0.92)	0.91 (0.90–0.92)

a ROC-AUC evaluation on the unseen test set for the classification of protein existence, with a 95% confidence interval (lower, upper) estimated using the DeLong method.

### 3.2 Model new prediction evaluation

#### 3.2.1 Additionally identified proteins from unconfident proteins

For the evaluation of unconfident protein prediction in the sample, we focused on proteins that were not used in the training process, which were unlabeled proteins whose existence cannot be decided by pooled evidence from replicates.

For the gastric cell dataset, we categorized proteins as unconfident if their raw probability was below 0.9, a threshold chosen to approximately align with the conventional 5% global FDR level applied in protein identification, which resulted in 7466 proteins.

The ranking of predicted probability was more meaningful than the absolute value in deciding additionally identified proteins from unconfident proteins since it controlled which proteins were selected given a fixed number of proteins preferred to be added. The predicted and raw probabilities were sorted in descending order, and the top N proteins were selected as additional identified proteins.

**Figure 3.**
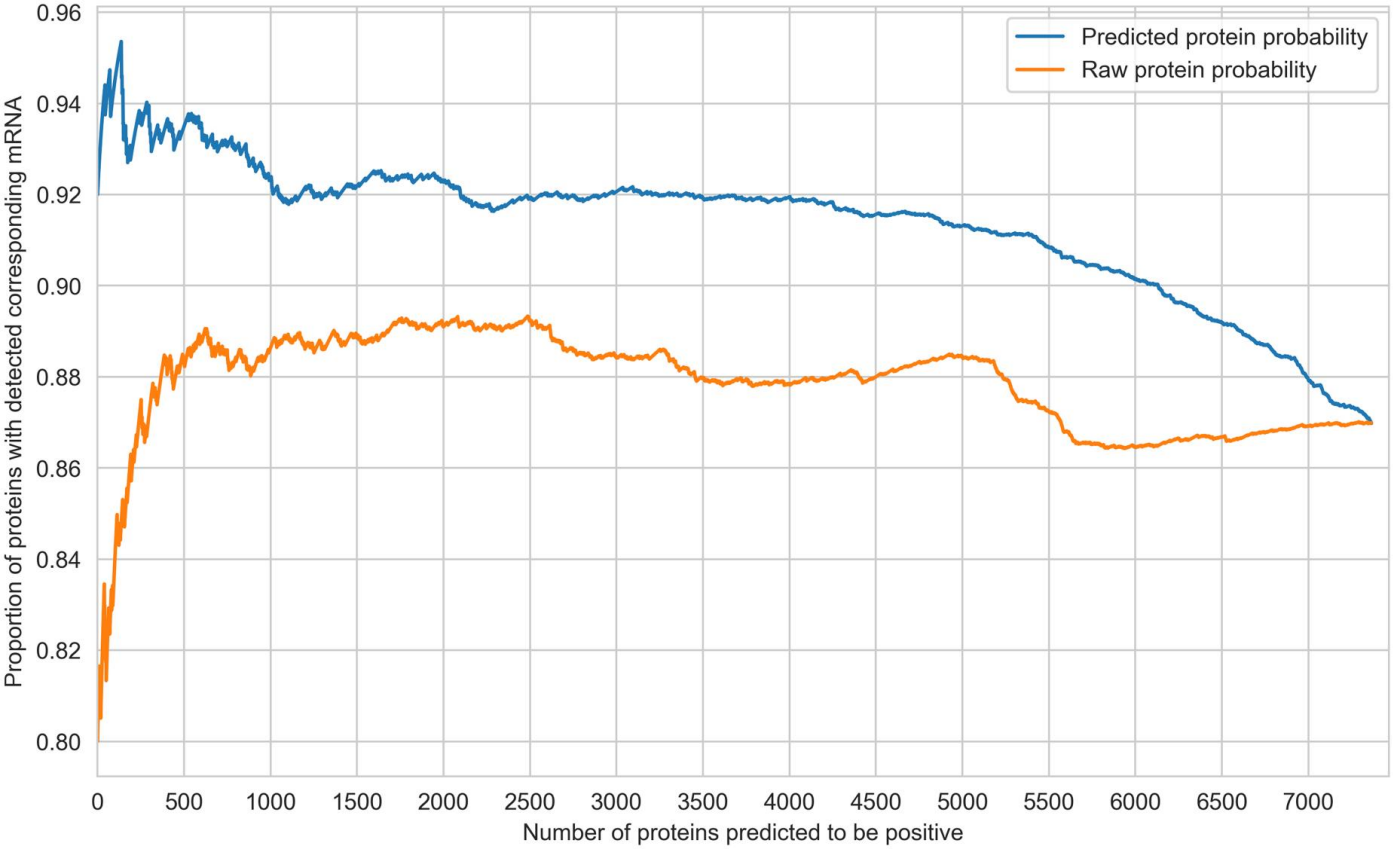
Proportion of proteins with detected corresponding mRNA. The *X*-axis represents the number of additionally identified proteins from unconfident proteins (defined as with raw probability less than .9) when using predicted probability and raw probability when considered in descending order (sequentially promoting more proteins based on their extent of evidence). This proportion was constantly higher for proteins identified by predicted probability.

According to the central dogma of molecular biology, every protein in a sample should have the corresponding mRNA from which it is translated. Therefore, assessing the mRNA coverage rate, which is the proportion of proteins accompanied by their corresponding mRNA, serves as an empirical measure for verifying the quality of proteins additionally identified within a sample. This premise was substantiated through the observed higher values of mRNA coverage rate for confidently identified proteins, which were proteins using a cutoff for raw probability <.9 (Supplementary Fig. S4). The mRNA coverage rate for additionally identified proteins by predicted probability was constantly higher than those proteins identified as proteins by raw probability, and the difference diminished gradually as more proteins were selected, and the overlap between additionally identified proteins from the two methods increased (Fig. 3). We also validate the model by applying a trained model from PCGC dataset to predict unconfident proteins in another cell-line gastric dataset from the latest Bruker timsTOF HT—the same pattern was also observed in this less unconfident protein scenario (Supplementary Fig. S13).

**Figure 4.**
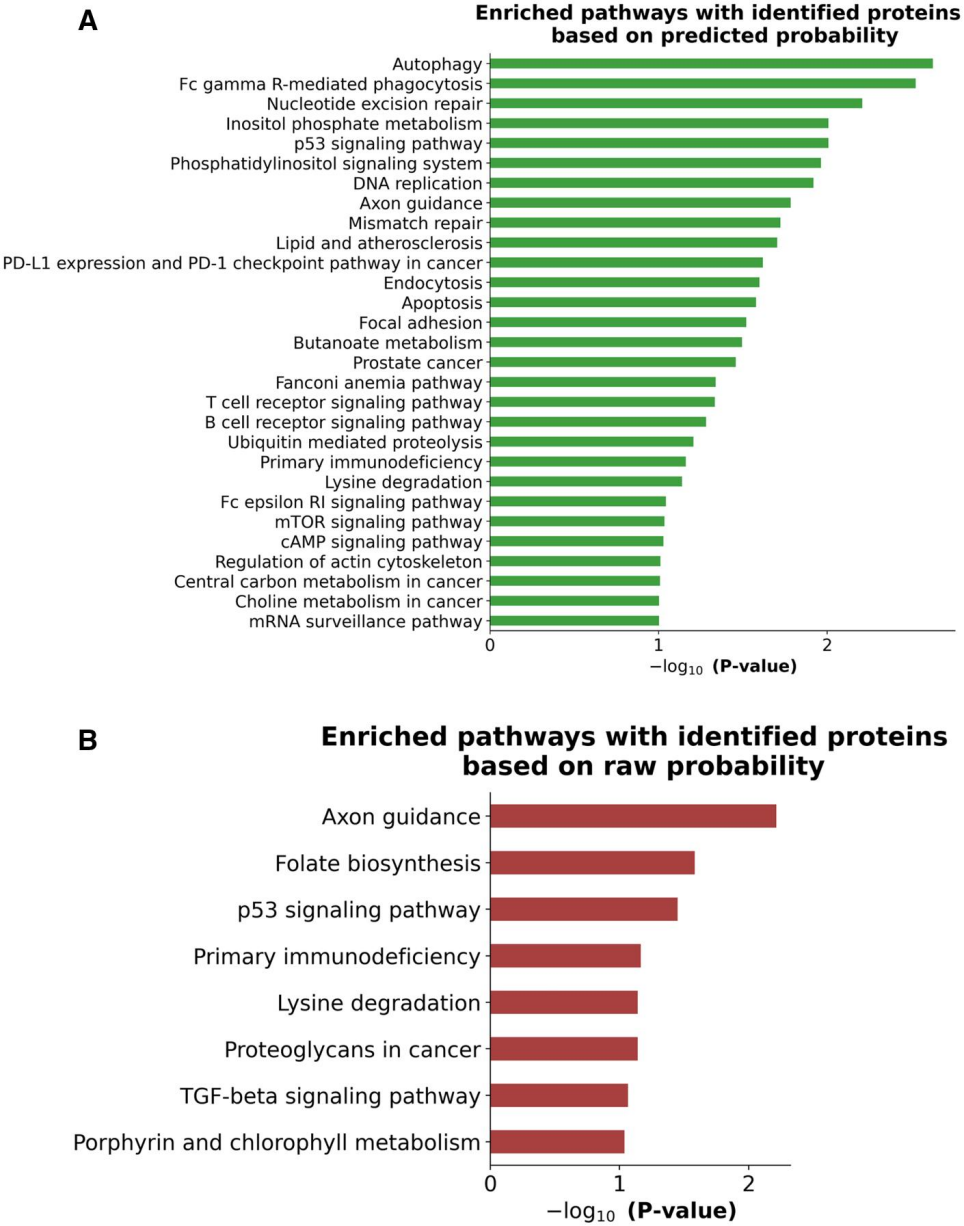
Pathway enrichment analysis from Grape-Pi model. The pathway analysis used 500 additionally identified proteins based on Grape-Pi predicted probability. (A): top enriched KEGG pathways from the 500 additionally identified proteins based on the Grape-Pi predicted probability with *P*-value < .1. (B): top enriched KEGG pathways from the 500 additionally identified proteins based on raw probability with *P*-value < .1.

At the cutoff of 500 (500 additional proteins being detected), the proportion of proteins being detected with corresponding mRNA is 93% using Grape-Pi-GCNConv method versus 88% for raw probability selection method. Notably, of the 500 newly identified proteins from predicted probability, a large portion (219/500) were not identifiable using raw probability at this desired number of additionally identified proteins. The pathway enrichment analysis performed with 500 identified proteins with the Grape-Pi method yielded enrichment of cancer-related pathways in gastric samples (Fig. 4).

#### 3.2.2 Interpretation of model decision

**Figure 5.**
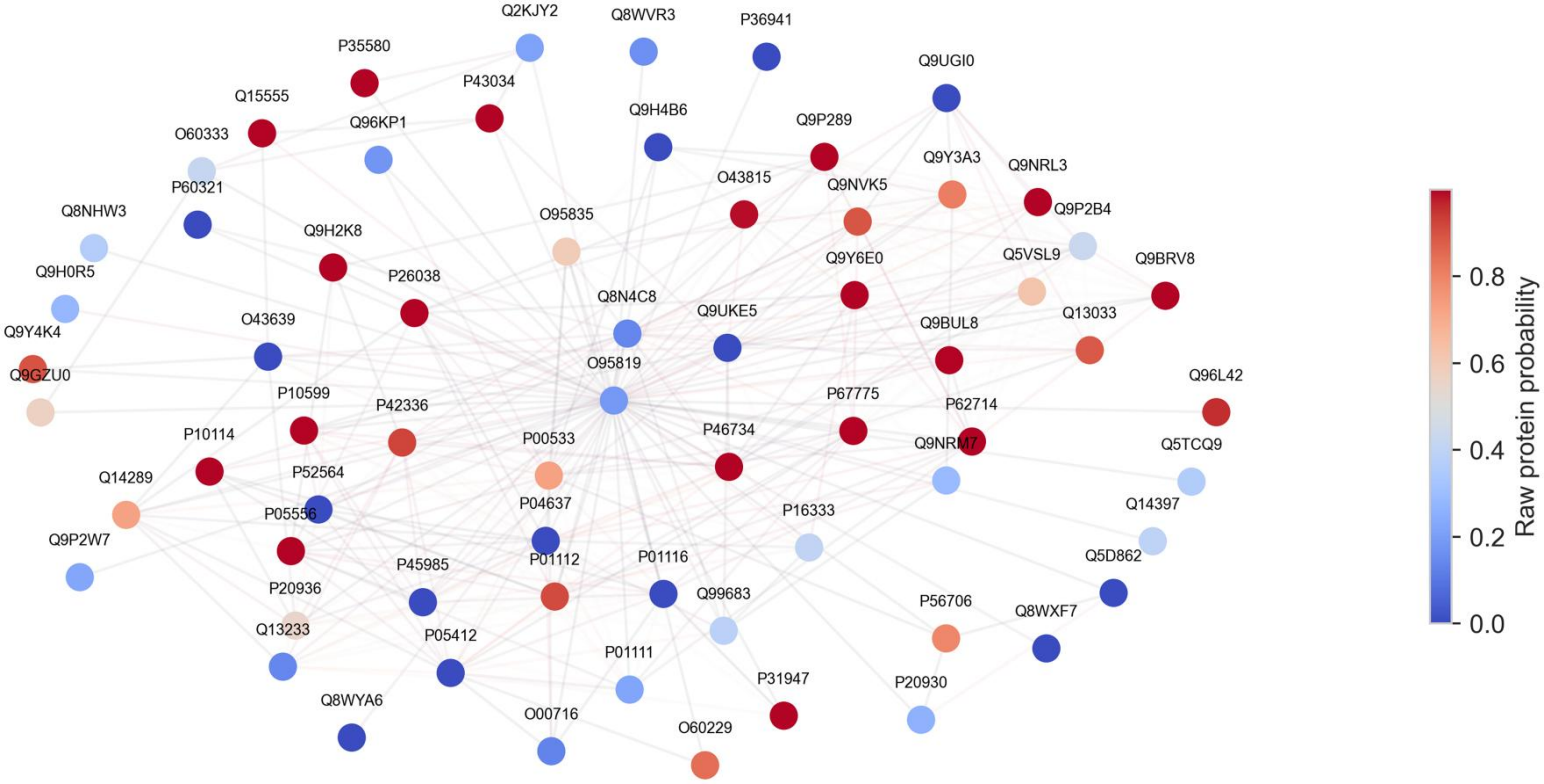
Illustration of model decision for promoted protein. Subnetwork within one hop from the center protein O95819 (gene: *MAP4K4*). The same figure with gene symbols as node labels can be found in Supplementary Fig. S7.

**Figure 6.**
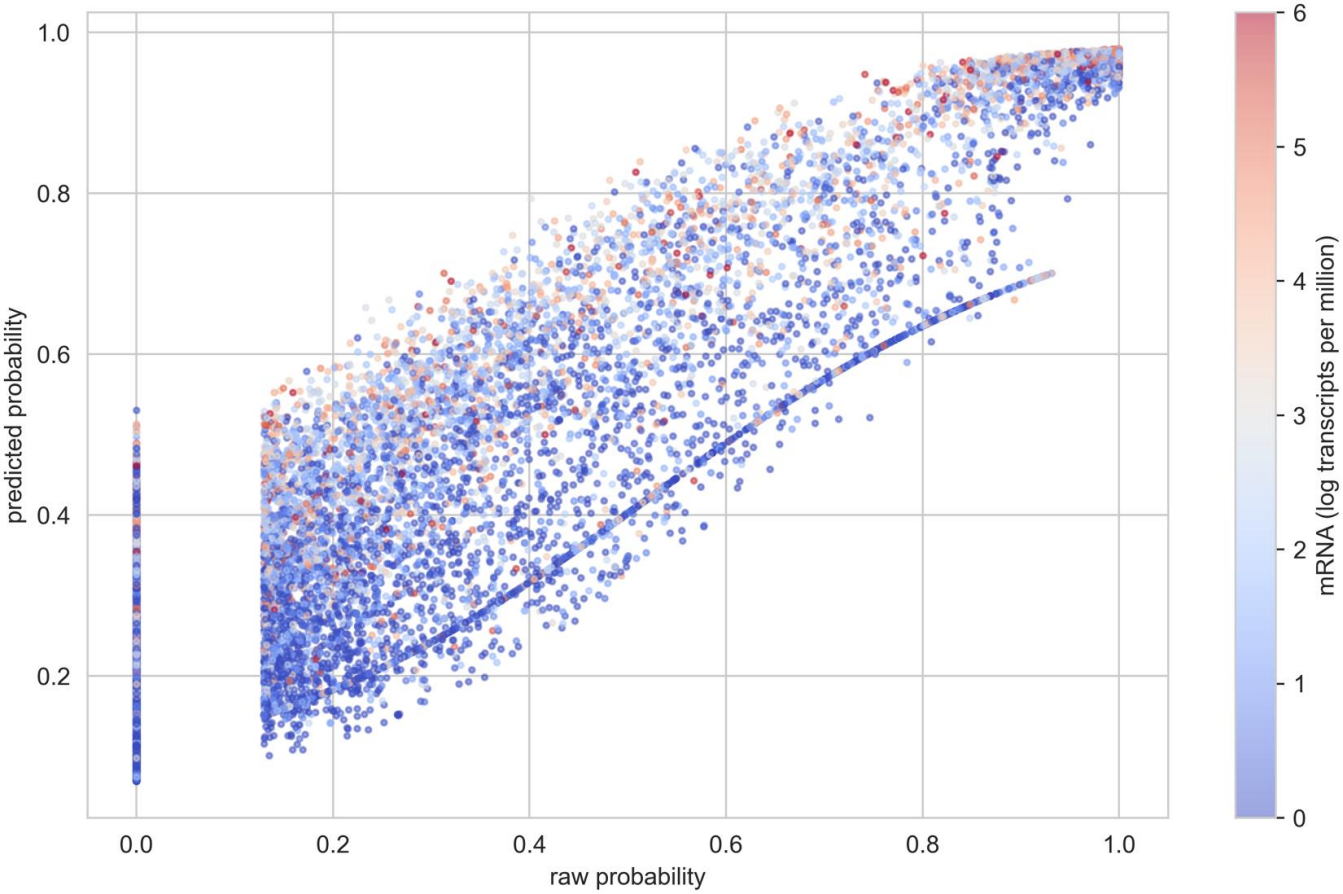
Scaling effects of the Grape-Pi model. The scatter plot illustrates the upscaling and downscaling of raw probabilities to predicted probabilities. The color represents the mRNA expression in the log scale. High-confidence proteins remain largely unaffected, while upscaled proteins exhibit higher corresponding mRNA expression levels.

Interpreting the decision of deep learning remains challenging, particularly with high-dimensional and non-structured data. To examine whether the model aligned with its intended purpose, we determine protein existence through the integration of information from itself and interacting proteins. We exemplified it with a promoted protein (UniProt O95819, gene *MAP4K4*), which was identified as a potential biomarker for late-stage gastric cancer and other cancers in previous studies (Zhang *et al.* 2022, Chen *et al.* 2023). While the protein was initially identified with a raw probability of .19 (rank 3182 in unconfident proteins), it had a predicted probability of .54 and a rank of 195 in our new prediction approach. Subnetworks centered on the example protein were constructed to illustrate the model decision mechanism. The interacting proteins surrounding the promoted protein showed an apparent positive pattern (Fig. 5). We analyzed the distribution of predicted probabilities across different regions of raw probabilities and observed that high-confidence proteins remain largely unchanged. In contrast, proteins with upscaled probabilities show higher corresponding mRNA expression levels (Fig. 6). A full list of new predicted scores versus raw protein probabilities is shown in the Supplementary File. An example of a demoted protein can be found in Supplementary Fig. S10.

### 3.3 Additional information as node features

The model performance can be further improved by incorporating additional protein information as node features. Integrating mRNA expression with raw probability has also been shown to be effective in improving protein identification (Ramakrishnan *et al.* 2009). Using the same model design and hyperparameter but using mRNA expression in addition to the raw probability as additional features, the ROC AUC was boosted to 0.86, 0.88, and 0.94, respectively, for yeast-LCQ, yeast-ORBI, and gastric cancer sample (Supplementary Table S3). Other protein information, such as protein structure/function and protein-level mass spectrometry information from protein analysis software, could also boost the model’s performance further. It is worth noting that additional features may need to be carefully processed, such as encoding and transformation, and the model trained with those features may become platform-dependent.

## 4 Discussion

Accurately identifying proteins in proteomics involves challenges in differentiating true positives from false positives, often leading to a high FNR to ensure reliability. To address this, researchers integrate protein scores with additional data, such as predicted peptide detectability and PPI networks, though traditional models require strong assumptions and may not fully exploit the complex nature of protein interactions. Here, we proposed a GNN-based approach (Grape-Pi) that uses PPI information to improve protein detection accuracy. Our model showed ubiquitously improvement over samples from different species and MS instruments. However, the extent of improvement varied across different experimental settings and depending on many factors, such as the complexity of the sample, the quality of the original raw probability, the reliability of PPI data, and the accuracy of ground-truth labels for protein existence.

Compared with previous research that tried to model raw probability jointly with PPIs, our model is more flexible and data-driven, offering the capacity to incorporate rich protein node features such as mRNA expression, protein physical or chemical properties, function, and localizations, even high-dimensional embedding from protein large language model (Lee *et al.* 2020, Rives *et al.* 2021). These complex features are often difficult to use in traditional models but are efficiently processed by GNNs. Although one message-passing layer achieved the best performance, this is likely due to the STRING database’s comprehensive PPI network, which includes both direct physical interactions and indirect connections (e.g. co-expression, co-occurrence, and predictions). This broad integration reflects distant node information within immediate neighbors, reducing the need for additional layers. However, with a custom PPI network focused on direct interactions, multiple message-passing layers may become optimal and would be challenging to handle with simpler algorithms. Grape-Pi is designed as a flexible framework rather than a one-size-fits-all solution. Users can train models and fine-tune hyperparameters to optimize performance for various datasets. A recent study (Luo *et al.* 2024) found that classical GNN architectures outperform more complex models when hyperparameters are optimally tuned, emphasizing the importance of offering comprehensive hyperparameter options and user-friendly tuning capabilities provided within the framework. As sample species’ complexity increases, PPI networks’ coverage and quality generally degenerate. For coverage, Hart *et al.* (2006) estimated that only about 10% of human protein interactions were known compared with what is about 50% for yeast. This discrepancy aligns with the observation in this study, where 3660 proteins in the PCGC dataset did not have an interactor, hindering the improvement in their identification through PPI-based approaches, compared with only around 100 such proteins in the yeast-rich-medium dataset. Regarding the quality of the PPI network, the data for yeast exhibited a higher confidence compared to that of humans overall. Since all interactions were treated equally in this study, a more confident PPI network could more effectively guide the flow of information from the neighboring nodes to the target node, leading to a better performance in the case of yeast datasets. The reluctant efforts to refine PPI network coverage and quality are anticipated to bolster the efficacy of network-based proteomics approaches field (Li *et al.* 2009), particularly for Grape-Pi, which leverages the PPI network to its full potential. Due to the nature of deep learning, the new predicted probability should be used in relative order, with cutoff points established based on the desired number of additional proteins to be classified as positive. In most machine-learning approaches, FDR estimation is performed by ranking scores and determining the proportion of decoy proteins at each threshold. However, this approach is not straightforward for graph machine-learning approaches such as Grape-Pi due to the absence of PPI data for decoy proteins. A possible solution would be to empirically estimate the FDR by simulating a synthetic PPI network that closely mirrors the structure of a real network, thereby allowing decoy protein scores to be calculated. However, designing a reliable method to simulate such PPI networks requires further investigation in future research.

Transcriptomics data measured on the same sample or sample under comparable settings can be used to guide the number of additional proteins to be included, particularly at the point where mRNA coverage declines sharply. Nonetheless, alternative methods can also be used. One option is to include the same number of proteins based on predicted probability as would be included under a relaxed FDR (e.g. 5%). In addition, if specific target proteins are of interest but not detected using conventional methods, the investigator can examine their relative ranks in predicted probability. If a protein ranks highly, it may be conditionally accepted, with further validation to confirm its presence.

One fundamental component of the semi-supervised learning model lies in the accuracy of ground-truth labels. In the context of protein identification, it is about distinguishing the fingerprint of protein existence from the sample variation. We employed a robust mean-pooling approach to address this challenge within the PCGC dataset to create ground-truth labels. However, it is almost certain that there are still residuals of sample variation in the created “ground-truth” label. The new generation of antibody-based (e.g. Olink) and aptamer-based (e.g. SomaScan) high-throughput techniques can generate a reasonably large number of proteins with high specificity. However, studies have reported limited overlap in protein detection and quantification between these platforms (Rooney *et al.* 2023, Wang *et al.* 2025). Future research could explore integrative approaches that leverage multiple technologies to establish more robust ground-truth labels, thereby improving the training and evaluation of protein identification and inference frameworks.

Our approach was founded on the idea that proteins participating in the same biological process or pathway are likely to be located near each other within the protein interaction network, which aligns with enrichment analysis. Enrichment analysis using GO and KEGG can be viewed as a simplified representation of groups of PPIs. They categorize proteins based on shared functions, processes, or cellular components, which can reflect the underlying interactions among those proteins. To reduce the chance of false positives, we recommend using high-quality protein interactions, such as protein interaction from KEGG, to reduce the chance of false positives, and protein identification after model enhancement shall improve the separation between active and inactive pathways during downstream enrichment analyses.

Techniques such as match-between-runs (MBR) (Cox and Mann 2008) and the use of high-quality spectral libraries (Sim *et al.* 2020, Zhong *et al.* 2020) also leverage prior information to improve the identification of low-confidence proteins. Given the distinct prior employed by these methods, we believe that the Grape-Pi can be complementary and orthogonal to the existing approaches. Grape-Pi cannot yet directly get protein quantification for additionally promoted proteins. However, it is possible to revisit peptide-level data and assign quantification values by using peptide intensities that were previously ambiguous across multiple proteins. Identifying the optimal strategy for deriving such values remains an open question for future research.

Finally, Grape-Pi, as a subtype of network-based proteomics approaches, is restricted to biological specimens where protein interaction networks are presumed to exist. Consequently, its efficacy may be limited in samples like plasma. It is particularly suited for salvaging protein in shotgun proteomics, where the FNR is sacrificed for false discovery control. However, for targeted proteomics, caution is warranted in scenarios where the FNR is a significant issue (Li *et al.* 2009). It is noteworthy that proteins with high confidence showed a positive correlation with the number of interactors (node degree; Supplementary Fig. S8), a factor that was not evaluated in previous PPI-based approaches. One explanation is that highly connected (“hub”) proteins, which are more frequently detected and studied, tend to possess richer PPI profiles (Gillis *et al.* 2014). As a result, models utilizing PPI data may inherently amplify this effect, as proteins with more neighbors are more influential. This highlights the importance of using a well-curated PPI network to guide the model toward biologically relevant proteins.

In summary, Grape-Pi can incorporate comprehensive PPI and protein information beyond the scope of earlier models. It demonstrated the potential of GNN in enhancing protein identification in shotgun proteomics for the first time. Similarly to its predecessor (Li *et al.* 2009), Grape-Pi is platform-independent, offering the flexibility to complement outputs from any proteomics analysis software, such as commonly used ProteinProphet and Percolator. The software and code accompanying this study enable straightforward customization and training of the model.

## Supplementary Material

vbaf095_Supplementary_Data

## Data Availability

Software, application demo, and source code are available at: https://github.com/FDUguchunhui/Grape-Pi. Command line software can be installed through PyPi: https://pypi.org/project/grape-pi. The data underlying the article are available on GitHub at https://github.com/FDUguchunhui/GrapePi and Zenodo at https://zenodo.org/records/11310518. The PCGC raw mass spectrometry data are available at the Proteomics identification database (PRIDE) with accession PXD047203.
