# Supplementary material for: Grape-Pi: graph-based neural networks for enhanced protein identification in proteomics pipelines
Source: Bioinform Adv. 2025 Apr 26;5(1):vbaf095. doi: 10.1093/bioadv/vbaf095 (PMC12096076; doi:10.1093/bioadv/vbaf095)
Supplement: vbaf095_Supplementary_Data [file vbaf095_supplementary_data.zip › supplementary methods.docx]

Supplementary biology methods

**Patient cohort and sample collection**

A total of 8 gastric ascites cancer (GAC) patients with documented primary cell (PC) were selected for this study. All patients were treated at The University of Texas MD Anderson Cancer Center (Houston, USA). PC specimens were obtained during a therapeutic procedure once patients provided a written approved informed consent document under an Institutional Review Board-approved protocol. PC is stage 4 cancer under the American Joint Committee on Cancer Staging Manual (8th edition)(Ajani, Lee et al. 2017, Siegel, Miller et al. 2018). The clinical characteristics and treatment information of these patients are listed in Supplementary Table 1. Information regarding patient treatment was provided in Supplementary File 3.

The collection of PC cells was as described previously(Ajani, Xu et al. 2021). Ascites samples ranging from 100 mL to 2000 mL were obtained from GAC patients who underwent a therapeutic and/or diagnostic procedure. Cells were spun down at 2,000g for 20 minutes. Pelleted cells were lysed in red blood cells lysis buffer consisting of 0.079 g of ammonium bicarbonate and 6.1 g of ammonium chloride in 1 L of distilled H2O. Red blood cells were removed to enrich PC cells for further analysis. The percentage of malignant cells was recorded by a pathologist or cytologist.

Generation of mass spectrometry-based proteomic data and analysis

Proteomic analysis of gastric cancer primary cell sample was performed using mass spectrometry as previously described(Katayama, Tsou et al. 2019, Capello, Katayama et al. 2022, Fahrmann, Tanaka et al. 2022). Total cell extracts (TCEs) were obtained by sonication of cell lysate pellets in 4M Urea, 3% IsoPropanol, 20 mM Tris containing the detergent 2% octyl-glucoside (OG, Sigma-Aldrich) and protease inhibitors (complete protease inhibitor cocktail, Roche Diagnostics) and phosphatase inhibitor (PhosSTOP, Roche Diagnostics) followed by centrifugation at 20,000 x g at 4 °C for 30 min.

Technology improvement in the ion-mobility-assisted data-independent acquisition (DIA) method has been shown to be beneficial in covering the depth of heterogeneous clinical samples because of the additional gas phase separation and unbiased parent ion fragmentations. We further applied protein level HPLC fractionation followed by trypsin digestion to de-complex the peptides up-front, and continuously analyzing the samples by ion-mobility LC-MS/MS (DIA) enabled us to dig deeper, especially on those low input clinical samples.

TCE proteins were reduced in DTT and alkylated with acrylamide before the fractionation of intact proteins using RP-HPLC. Proteins were desalted and separated using an off-line AQUITY UPLC Class-H system (WATERS) with a reversed-phase column (4.6-mm internal diameter × 150-mm length; Column Technology). Collected fractions were dried by lyophilization followed by in-solution digestion with trypsin (Mass Spectrometry Grade, Thermo Fisher).

A total of 23 fractions were subjected to ion-mobility LC-MS/MS (DIA: Data Independent Acquisition) analysis per ascites sample. Tryptic peptides were injected onto reversed phase nanoLC-MS/MS using a nanoAquity LC system coupled online with SYNAPT G2-Si ion-mobility mass spectrometer (WATERS). Separations were performed using 75 µm id × 360 µm od × 25-cm-long fused-silica capillary column (Column Technology) slurry packed with 3 µm, 100 A° pore size C18 silica-bonded stationary phase. Following injection of ~ 2 µg of protein digest onto a C18 trap column (Waters, 180 µm id × 20 mm), peptides were eluted using a linear gradient of 0.35% mobile phase B (0.1 formic acid in ACN) per minute for 90 min, then to 95% B in an additional 10 min, all at a constant flow rate of 300 nL/min.

LC-HDMSE Data was acquired in high-resolution mode using Waters Masslynx (version 4.1, SCN 851). The capillary voltage was set to 2.80 kV, sampling cone voltage to 30 V, source offset to 30 V, and source temperature to 100 °C. Mobility utilized high-purity N2 as the drift gas in the IMS TriWave cell. Pressures in the helium cell, Trap cell, IMS TriWave cell, and Transfer cell were 4.50 mbar, 2.47e-2 mbar, 2.90 mbar, and 2.53e-3 mbar, respectively. IMS wave velocity was 600 m/s, helium cell DC was 50 V, Trap DC bias was 45 V, IMS TriWave DC bias was 3 V, and IMS wave delay was 1,000 µs. The mass spectrometer was operated in V-mode with a typical resolving power of at least 20,000. All analyses were performed using positive mode ESI using a NanoLockSpray source. The lock mass channel was sampled every 60 s. The mass spectrometer was calibrated with a [Glu1] fibrinopeptide solution (300 fmol/µL) delivered through the reference sprayer of the NanoLockSpray source. Accurate mass LC-HDMSE data was collected in an alternating, low energy (MS), and high energy (MSE) mode of acquisition with a mass scan range from m/z 50 to 1800. The spectral acquisition time in each mode was 1.0 s with a 0.1-s inter-scan delay. Data were collected in the low energy HDMS mode at a constant collision energy of 2 eV in both the Trap cell and Transfer cell. In high-energy HDMSE mode, the collision energy was ramped from 25 to 55 eV in the Transfer cell only. The RF applied to the quadrupole mass analyzer was adjusted such that ions from m/z 300 to 2,000 were efficiently transmitted, ensuring that any ions observed in the LC-HDMSE data less than m/z 300 were known to arise from dissociations in the Transfer collision cell.

The acquired LC-HDMSE data were processed and searched against the Uniprot proteome database (Human, January 2017) through ProteinLynx Global Server (PLGS, Waters Company) with two trypsin miss cleavage allowed. The modification search settings included cysteine (Cys) alkylation with acrylamide (71.03714@C) as a fixed modification and methionine (Met) oxidation (15.99491@M) as a variable modification. The searched data was filtered with a False Discovery Rate 4%. The spectral counts for each protein driven by the identified peptides were used as a measure of protein abundance. The normalization was done by each protein's spectral counts divided by the total spectral counts of each patient ascites cells multiplied by the factor 100,000.

**DNA and RNA data analysis**

Whole-exome sequencing (WES) was performed on 22 of 26 PC specimens, with 11 having matched gDNAs from peripheral blood. RNA sequencing was performed on 21 specimens for which MD data were available. DNA and RNA extractions, sequencing, data processing, quality check, and analysis were performed as previously described^7^.

**Proteomics Platform using Bruker instrument:**Proteomics analyses will be conducted by LC-MS/MS on a EvoSep nano-LC coupled timsTOF HT mass spectrometry (Bruker). The timsTOF HT is the newest generation, state-of-art technology by Bruker that allows trapped ion-mobility spectrometry (tims) gas phase separation during the LC-MS/MS runs, that reduces requirement for pre-fractionation of precious clinical samples thus improving sample throughput, while simultaneously providing unprecedented increases in sensitivity, dynamic range and the depth of the protein characterization.

# Supplementary Methods

## Protein dataset structure

The graph is a type of unstructured data, and our particular “protein” dataset class requires protein information from multiple sources to be built for training the model, including ground-truth labels of existence for proteins in the sample, protein-protein interactions (PPI), protein features from MS instrument and other sources.

## Data labeling

The ground-truth label is a crucial component for training supervised and semi-supervised deep learning models by evaluating model prediction by quantifying deviations from the truth through a loss function. The deviation of current predictions from the truth guides subsequent model improvements, typically automated through gradient descent, and is repeated until the error is minimized or reaches a local minimum) error. The process of creating ground-truth labels for the data is known as data labeling.

Determining the ground-truth existences (labels) for proteins in a sample is challenging as only about 30% proteins are identified with sufficient confidence. The number of confident-identified proteins can vary with changes in sample composition, sample preparation methods, different MS device settings, searching algorithm, etc.

**Protein reference set**

The protein reference set is composed of two categories: proteins confidently identified as existent (positive proteins) and proteins considered confidently as non-existent (negative proteins). The protein reference set can be used to create the ground-truth label for the data. A commonly used approach for making a protein reference set was to run a sample under the same experiment setting multiple times (i.e., technique replicates) and classifying proteins that pass a certain reliability threshold (usually 5% FDR) in at least N experiments (where N typically represents half of total number of experiments) as positive proteins. Similarly, negative proteins are those that pass the threshold in less than M instances (usually M is 1, indicating no confident identification in any experiments). When technique replicates are unavailable, data from biologically similar samples subjected to comparable experiment settings (i.e., biology replicates) can be used. This approach was used for creating labeling for yeast-rich-medium datasets (yeast-LCQ and yeast-ORBI) in Ramakrishnan, Vogel et al. (2009). We have adopted these two yeast datasets as benchmark datasets to demonstrate the effectiveness of our model.

However, the label creation through the approach above could be subjective due to the arbitrary choices of threshold N and M especially. It also suffered from significant variability in a model’s results with different selection of N and M, especially when the total number of replicates is limited. Thus, we introduced a more robust labeling approach in this study: the protein confidence probability for each protein was acquired by mean pooling protein confidence probability from technique or biology replicates (See Supplementary Methods). Then, a protein was considered positive if the pooled protein probability was greater than 0.7 and negative if less than 0.3, and all other proteins were left unlabeled. This labeling approach provided a smoother adjustment of the threshold when the number of replicates used for creating labels was limited. W applied this labeling approach to the primary cell gastric cancer (PCGC) dataset, and more details about how labels were created were provided in section 3.2.

## Protein-Protein Interaction Data

PPI database contains details about the interaction between pairs of proteins, including the interaction type between two proteins (physical or functional) and evidence score. This information was used to build an adjacency matrix and edge feature matrix (or edge attribute matrix) for the protein dataset. The adjacent matrix in the protein dataset is matrix where the rows and columns represent individual proteins in the protein network. The value of one indicates the presence of an interaction between a pair of proteins, while a zero indicates no interaction. The Edge feature matrix represents attributes for edges, with each row corresponding to an edge and columns corresponding to different features associated with the edges. The adjacent matrix and edge feature matrix are additional information that other deep learning architectures do not usually consider. Thus, a reliable source of PPI data is crucial for GNN models to perform better than the other models.

STRING (v12.0) is a protein-protein interaction database that collects, scores, and integrates publicly available sources of interaction information to provide a comprehensive and objective global network of protein interactions (Szklarczyk, Gable et al. 2019), and we used it as our source of PPI information in this study.

The PPI dataset downloaded from STRING contained additional metadata about the interaction relationship between two proteins, such as confidence, strength, and type of interaction. This detailed information holds potential to further improve the model performance with meticulous preprocessing. However, for the purpose of our study, we focused solely on the binary relationships, specifically whether there is an interaction existed between two proteins. For better protein identification reliability, only interactions with a minimum combined confidence score of 0.4 were included.

The STRING used provided an overall combined confidence score for each interaction ranging from 0.15 to 0.999 (lower confidence: ≥ 0.15, medium confidence: 0.4, high confidence: ≥ 0.7, and highest confidence: ≥ 0.9). We tested filtering at several thresholds for smaller but more confident PPI data and found a slight improvement in the PCGC dataset at some thresholds. The choice of threshold depends on several factors from our experiment, including interaction dataset reliability and the size of the protein dataset. Besides the overall combined confidence score, STRING also provides confidence scores from each source (such as co-expression, experimental, and text mining), and using only interactions with co-expression evidence also slightly improved model performance. Those adjustments may serve as additional hyperparameters for fine-tuning the model. In the context of disease-specific studies, leveraging a PPI dataset tailored to the particular disease of interest could be more advantageous than using a general species PPI dataset.

### Protein feature data

The Protein feature data were used to create the node feature matrix, a matrix with each row corresponding to a protein and columns corresponding to features associated with the proteins. Protein features can be protein MS features from protein analysis software (e.g., ProteinProphet) and protein properties from public protein databases (e.g., ProteinAtlas). The most important feature of this data was the raw protein probability (raw probability for short), representing the protein existence confidence from a protein-level analysis software based only on MS experiments. Most modern protein software can directly output the raw probability; if not, it can be easily calculated based on the protein score in other forms (See Section 3.2).

## Models

We evaluated two different GNN message-passing layers for this task and evaluated their performance.

### Model 1: Grape-Pi-GCNConv

GCNConv was a spectrum-based graph convolution approach from the Graph Convolutional Network Field (Kipf & Welling, 2016). The idea was similar to convolution in image data. Spectral convolution on graphs is defined as the multiplication of the signal $X\in R^{N\times C}$ in the Fourier domain (N is the number of nodes, and F is the number of features for each node). The GCN model made several approximations that simplified the computation complexity to achieve fast localized convolution and introduced a renormalization trick for the exploding/vanishing gradient problem. The compact form of a GCN convolutional layer is defined as:

$$H=\tilde{D}^{-\frac{1}{2}}\tilde{A}\tilde{D}^{-\frac{1}{2}}\mathrm{XW}$$

Where $X\in R^{N \times C}$is the input matrix, $W\in R^{C\times C^{'}}$is the weight matrix with C input features and $C^{'}$ output features. $\tilde{A}=A+I_{N}$ and $\tilde{D}_{ii}=\sum j\tilde{A}_{ij}$ where A is the adjacency matrix. $\tilde{A}$ can be considered as the modified adjacency matrix with self-connection, and $\tilde{D}$ is the modified degree matrix (Kipf and Welling 2016).

A single GCN graph convolutional layer will aggregate information for a node from its immediate neighbor (first-order neighbor). Multiple such graph convolutional layers can be stacked to achieve $K^{th}-\mathrm{order}$neighborhood aggregation (the label of a node depends on nodes that are at maximum K steps away from it).

A simple two-layer GCN node classification model can be expressed as:

$$Z=f\left( X,A \right)=\mathrm{softmax}\left( \hat{A} ReLU\left( \hat{A} X W^{\left( 0 \right)} \right)W^{\left( 1 \right)} \right)$$

Where $\hat{A}=\tilde{D}^{-\frac{1}{2}}\tilde{A}\tilde{D}^{-\frac{1}{2}}$ is calculated in a pre-processing step. $W^{\left( 0 \right)}\in R^{C\times H}$ is the input-to-hidden weight matrix for a hidden layer with in-dimension  $C$ and out-dimension $H$. $W^{\left( 1 \right)}\in R^{H\times F}$ is the hidden-to-ouput weight matrix layer with in-dimension  $H$ and out-dimension $F$. The softmax activation function, defined as $\text{softmax}\left( x_{i} \right)=\frac{1}{Z}\exp\left( x_{i} \right)$ with $Z=\sum_{i} \exp\left( x_{i} \right)$. For semi-supervised multiclass classification, the loss is set as the cross-entropy error over all labeled examples:

$$\mathcal{L=-}\sum_{l\in\mathcal{V}_{\mathcal{L}}} \sum_{f=1}^{F} Y_{lf}\ln Z_{lf},$$

where $y_{L}$ is the set of node indices that have labels.

The formula can be easily extended to a K-layer GCN model (Kipf and Welling 2016).

Spectrum-based graph convolution models such as Grape-Pi-GCNConv are transductive, which means that the entire graph structure must be fixed and used during training, which includes connectivity information of nodes from validation and test datasets. Transductive models trained on a dataset cannot be generalized to another dataset without non-trivial changes.

### Model 2: Grape-Pi-SAGEConv

The second model was chosen to represent the spatial graph convolution approaches. The message passing layer used was from GraphSAGE (Graph Sample and aggreGatE).

GraphSAGE uses a different strategy for aggregating neighborhood information for a node. It samples a fixed-size set of neighbor nodes from each depth and uses trainable aggregation functions to aggregate features across its local neighborhood.

Due to its inductive nature, the GraphSAGE model can be easily generalized to streaming data and multi-graph settings, in which the model is trained with several different graphs, and predictions are made for nodes in other new graphs (nodes in the new graphs can be completely unlabeled). It is an interesting property in this protein identification task setting since biology replicates are usually available for a given experiment setting (For example, we may have MS data in breast cancer tissue samples from different patients). The usage of biology replicates can significantly expand the size of available training datasets, and more trainable parameters can be included to unravel the power of deep learning models.

## Experimental set-up

To identify the optimal hyperparameter configuration across a vast parameter space, we used GraphGym, a powerful platform for exploring different GNNs and tasks with modularized GNN implementation, standardized GNN metrics, and support for parallel experiments. GraphGym enhances scalability and reproducibility by setting up experiments through configuration files (You, Ying et al. 2020).

The GNN models in GraphGym comprise three crucial architecture design aspects: intra-layer design, inter-layer design, and learning configuration (Figure 1 Stage 2). We briefly introduced the options we have in each component, and for details about component options, please refer to the GraphGym Paper (You, Ying et al. 2020).

The intra-layer design configures the message-passing layer (the GNN layer). A message-passing layer defines the type of GNN model, including choices between GCNConv and SAGEConv layers, as well as the choices of whether to use batch normalization, dropout layers, and activation functions. The inter-layer design determines how layers are organized within neural network models. Layer connectivity specifies how GNN layers are connected, including stack, residual connection SKIP-SUM, and dense connection SKIP-CAT. Pre- /post-process layers were multi-layer perceptron (MLP) before/after the GNN message-passing layers that serve the purpose of feature selection and feature extraction. The training configuration included batch size, learning rate, optimizer type, and training epochs.

The design space of a specific type of GNN can be explored using a grid search. However, the complete design space would be enormous if we considered all possible combinations (~10M). Therefore, we fixed certain hyperparameters, including ReLU for the activation function and Adam as the optimizer, based on their consistently good performance across a wide range of deep learning tasks and architectures (Diederik and Ba 2017, Abien 2019). This allowed us to focus on a select group of hyperparameters for a comprehensive grid search (Table 1). Each combination was evaluated three times using random number seeds. The dataset was randomly split into 60/20/20 partitions for training, validation, and testing. Weighted cross-entropy loss was used to adjust for class imbalance between labeled positive and negative proteins.

We conducted batch experiments on both GrapePi-GCNConv and GrapePi-SAGEConv using the yeast-ORBI data, selecting the best models based on their performance in the validation dataset.

We evaluated the performance of our best GrapePi-GCNConv and GrapePi-SAGEConv on the test dataset for both yeast-LCQ and yeast-ORBI datasets. As a baseline comparison, we used three-layer multi-layer perceptrons (MLPs) with a similar number of parameters and, when possible, similar hyperparameter configurations.

## Supplementary analysis

1. Sensitivity analysis for using different thresholds in the protein-protein-interaction dataset (Figure S6)

2. Sensitivity analysis for using different protein labeling criteria (Figure S5)

3. Consistency assessment for predicted probability between two yeast-rich-medium samples of similar conditions. (Figure S13)

4. Distribution of the raw probability in newly identified proteins from predicted probability (Figure S9).

5. Correlation between the predicted probability and mRNA expression (compared with the correlation between raw probability and mRNA expression) (Figure S12)

6. Applying a pre-trained model to improve gastric cell-line data from Bruker mass spectrometry instrument. (Figure S14): the model trained on the primary-cell gastric cancer (PCGC) dataset was applied to the cell-line data from the Bruker mass spectrometry instrument. Details about the biology and mass spectrometry parameters used in the Bruker data can be found in the Supplementary Biology Method. Similarly, in the PCGC dataset, we consider proteins with a raw probability of less than 0.9 but greater than 0.1 as unconfident proteins. For those unconfident proteins, we then ranked predicted probability and raw probability in ascending order. We checked their proportion with corresponding mRNA expression (mRNA coverage rate) when gradually increasing the number of newly identified proteins. A consistently higher mRNA coverage rate indicates a better detection power.

# The best hyperparameter used for model evaluation

## SAGEConv

*# The recommended basic settings for GNN*num_workers: 8
out_dir: results
metric_best: auc
dataset: *# need to delete file in processed folder if change args under dataset category* name: protein
 dir: data/gastric_all_data
 rebuild: true
 numeric_columns:
 - protein_probability
*# - mRNA_TPM* label_column: hard_label
 remove_unlabeled_data: false
 task: node
 task_type: classification
 transductive: false
 transform: none
 encoder: false
 node_encoder: false
 edge_encoder: false
 split: [0.7, 0.2, 0.1] *# train, val, test*share:
 dim_in: -1
 dim_out: 2 *# dim_out 2 is for binary classification*train:
 grape_pi: graphsage
 loss_pos_weight: -1.0
 batch_size: 128
 ckpt_period: 10
 ckpt_clean: false
 sampler: neighbor
 eval_period: 10
 neighbor_sizes: *# sample 20 from 1-order neighbor and so on* - 20
 - 10
 - 5
 epoch_resume: 0
model:
 type: gnn
 loss_fun: binary_cross_entropy_with_weight
 *# the weight is calculated by num_negative_sample/num_positive_sample*gnn:
 layers_pre_mp: 1
 layers_mp: 1
 layers_post_mp: 1
 dim_inner: 10
 layer_type: sageconv *# sageconv gcnconv* stage_type: skipsum *#stack skipsum skipconcat* batchnorm: false
 act: relu
 dropout: 0.0
 normalize_adj: false
 head: protein
optim:
 optimizer: adam
 base_lr: 0.001
 weight_decay: 5e-4
 max_epoch: 200
 *# try not use schedule and check fluctuation of training curve* scheduler: none

## GCNConv

*# The recommended basic settings for Grape-Pi*num_workers: 8
out_dir: results
metric_best: auc
dataset: *# need to delete file in processed folder if change args under dataset category* name: protein
 dir: data/single
 rebuild: true
 numeric_columns:
 - protein_probability
 - mRNA_TPM
 task: node
 task_type: classification
 transductive: true
 transform: none
 encoder: false
 node_encoder: false
 edge_encoder: false
 split: [0.6, 0.2, 0.2] *# train, val, test*share:
 dim_in: -1
 dim_out: 2 *# dim_out 2 is for binary classification*train:
 grape_pi: gcnconv
 loss_pos_weight: -1.0
 batch_size: 1
 ckpt_period: 10
 ckpt_clean: false
 sampler: full_batch
 epoch_resume: 0
model:
 type: gnn
 loss_fun: binary_cross_entropy_with_weight
 *# the weight is calculated by num_negative_sample/num_positive_sample*gnn:
 layers_pre_mp: 1
 layers_mp: 1
 layers_post_mp: 1
 dim_inner: 10
 layer_type: gcnconv *# sageconv gcnconv* stage_type: skipsum *# stack skipsum skipconcat* batchnorm: false *# batchnorm make things worse* act: relu
 dropout: 0.3
 normalize_adj: false
 head: protein
optim:
 optimizer: adam
 base_lr: 0.001
 weight_decay: 5e-4
 max_epoch: 300
 *# try not use schedule and check fluctuation of training curve* scheduler: none

Abien (2019). "Deep Learning using Rectified Linear Units (ReLU)." arXiv pre-print server.

Ajani, J. A., J. Lee, T. Sano, Y. Y. Janjigian, D. Fan and S. Song (2017). "Gastric adenocarcinoma." Nat Rev Dis Primers **3**: 17036.

Ajani, J. A., Y. Xu, L. Huo, R. Wang, Y. Li, Y. Wang, M. P. Pizzi, A. Scott, K. Harada, L. Ma, X. Yao, J. Jin, W. Zhao, X. Dong, B. D. Badgwell, N. Shanbhag, G. Tatlonghari, J. S. Estrella, S. Roy-Chowdhuri, M. Kobayashi, J. V. Vykoukal, S. M. Hanash, G. A. Calin, G. Peng, J. S. Lee, R. L. Johnson, Z. Wang, L. Wang and S. Song (2021). "YAP1 mediates gastric adenocarcinoma peritoneal metastases that are attenuated by YAP1 inhibition." Gut **70**(1): 55-66.

Capello, M., H. Katayama and S. M. Hanash (2022). "Proteomic Profiling of the Tumor Microenvironment." Methods Mol Biol **2435**: 157-167.

Diederik and J. Ba (2017). "Adam: A Method for Stochastic Optimization." arXiv pre-print server.

Fahrmann, J. F., I. Tanaka, E. Irajizad, X. Mao, J. B. Dennison, E. Murage, J. Casabar, J. Mayo, Q. Peng, M. Celiktas, J. V. Vykoukal, S. Park, A. Taguchi, O. Delgado, S. C. Tripathi, H. Katayama, L. M. S. Soto, J. Rodriguez-Canales, C. Behrens, I. Wistuba, S. Hanash and E. J. Ostrin (2022). "Mutational Activation of the NRF2 Pathway Upregulates Kynureninase Resulting in Tumor Immunosuppression and Poor Outcome in Lung Adenocarcinoma." Cancers (Basel) **14**(10).

Katayama, H., P. Tsou, M. Kobayashi, M. Capello, H. Wang, F. Esteva, M. L. Disis and S. Hanash (2019). "A plasma protein derived TGFbeta signature is a prognostic indicator in triple negative breast cancer." NPJ Precis Oncol **3**: 10.

Kipf, T. N. and M. Welling (2016). "Semi-Supervised Classification with Graph Convolutional Networks." arXiv.

Ramakrishnan, S. R., C. Vogel, J. T. Prince, Z. H. Li, L. O. Penalva, M. Myers, E. M. Marcotte, D. P. Miranker and R. Wang (2009). "Integrating shotgun proteomics and mRNA expression data to improve protein identification." Bioinformatics **25**(11): 1397-1403.

Siegel, R. L., K. D. Miller and A. Jemal (2018). "Cancer statistics, 2018." CA Cancer J Clin **68**(1): 7-30.

Szklarczyk, D., A. L. Gable, D. Lyon, A. Junge, S. Wyder, J. Huerta-Cepas, M. Simonovic, N. T. Doncheva, J. H. Morris, P. Bork, L. J. Jensen and C. V. Mering (2019). "STRING v11: protein-protein association networks with increased coverage, supporting functional discovery in genome-wide experimental datasets." Nucleic Acids Res **47**(D1): D607-d613.

You, J., R. Ying and J. Leskovec (2020). "Design Space for Graph Neural Networks." arXiv pre-print server.

7 Jaffer Ajani, Shuangtao Zhao, Ruiping Wang et al. Proteogenomic Landscape of Gastric Adenocarcinoma Peritoneal Metastases, 05 October 2020, PREPRINT (Version 1) available at Research Square [https://doi.org/10.21203/rs.3.rs-81037/v1]
