# Supplementary material for: Grape-Pi: graph-based neural networks for enhanced protein identification in proteomics pipelines
Source: Bioinform Adv. 2025 Apr 26;5(1):vbaf095. doi: 10.1093/bioadv/vbaf095 (PMC12096076; doi:10.1093/bioadv/vbaf095)
Supplement: vbaf095_Supplementary_Data [file vbaf095_supplementary_data.zip › Supplementary Tables and Figures.pdf]

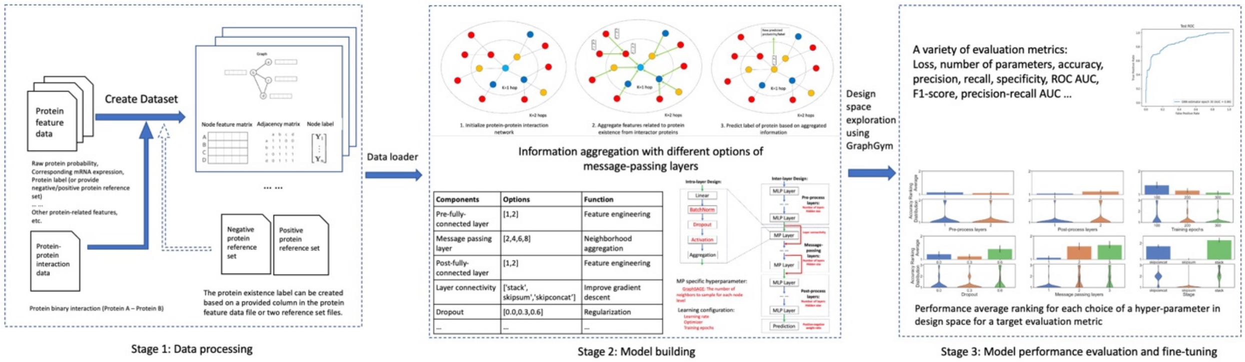

Figure 1. Flowchart Model training workflow: 1. Data processing: protein MS data and PPI data were first prepared and organized into a structured folder, and our protein dataset module loaded the data and converted them into a format that can be used to model training and model hyperparameter tuning. 2. Model building: Model building was aided with GraphGym by modularized design. The design space was explored guided by the grid search configuration file. 3. Ranking analysis was used to check which option for a hyperparameter was generally helpful using averaged ranking across all setups (see additional methods for more details). The best model based on the validation set was selected, and its performance on all datasets was evaluated using the hold-out test set of each dataset.

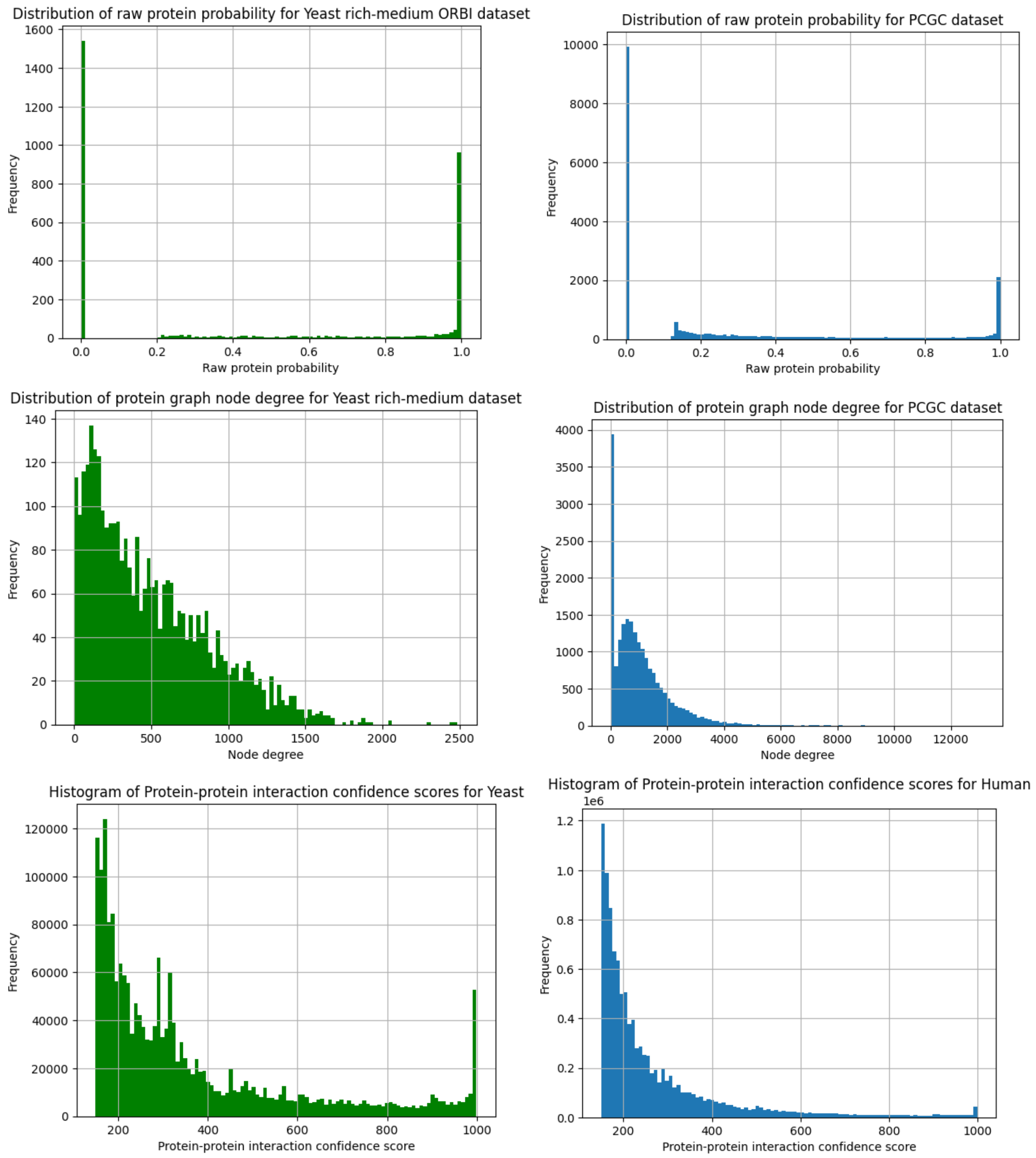

Supplementary Figure 2. Data properties of the Yeast and Primary cell gastric cancer (PCGC) graph dataset (Yeast on the left, and Human on the right). Raw protein probability for the Yeast dataset was similar to the PCGC dataset. There was a large part (3660) of proteins with zero interactors in the Human dataset. Excluding those proteins without interactor, proteins in the human dataset were more clustered (the number of interactors, a.k.a. node degree, were higher). Protein-protein interaction was more confident in the Yeast dataset than in the Human dataset.

Table S1a: Design space of Grape-Pi-GCNConv model

Dropout	Activation	Layer connectivity	Pre-process layers	Message passing layers	Post-process layers	Batch size*	Learning rate	Optimizer	Weight decay	Training epochs
0.0, 0.3, 0.6	ReLU	STACK, SKIP-SUM, SKIP-CAT	1, 2	1, 2, 3	1, 2	full_batch, 1	1e-4	Adam	5e-4	100, 200, 300

** Batch size for GCNConv model with only one graph will be 1, it means in each iteration, all nodes are used for forward and backward propagation.*

Table S1b: Design space of Grape-Pi-SAGEConv model

Dropout	Activation	Layer connectivity	Pre-process layers	Message passing layers	neighbor_sizes*	Post-process layers	Batch size	Learning rate	Optimizer	Weight decay	Training epochs
0.0, 0.3, 0.6	ReLU	STACK, SKIP-SUM, SKIP-CAT	1, 2	1, 2, 3	[20, 10, 5]	1, 2	64, 128, 256	1e-4	Adam	5e-4	100, 200, 300

** Neighbor_sizes hypermeter specifies how many nodes were sampled in each neighbor level.*

Best Models

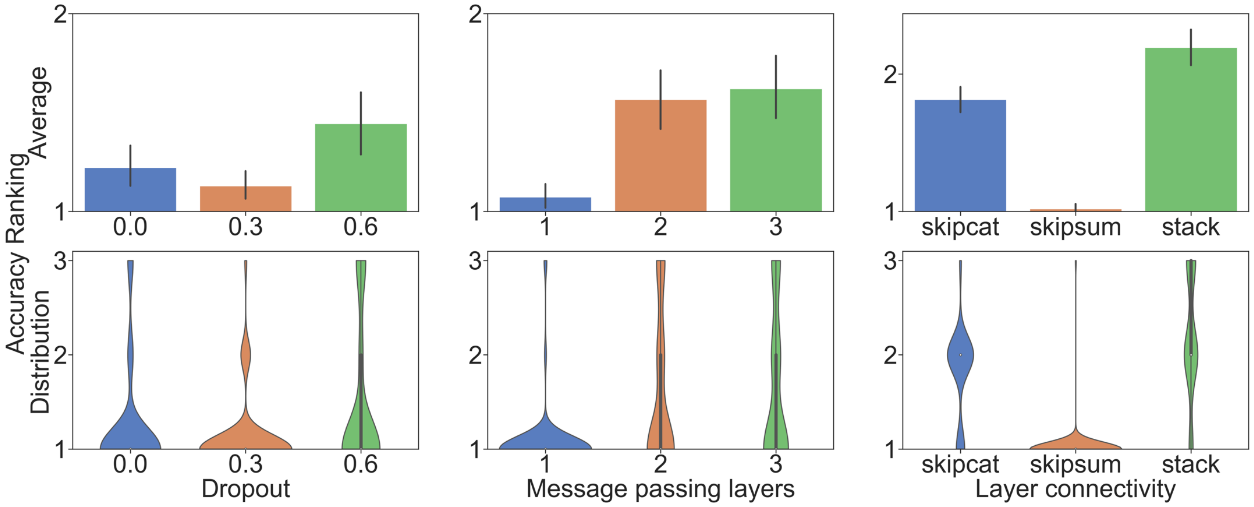

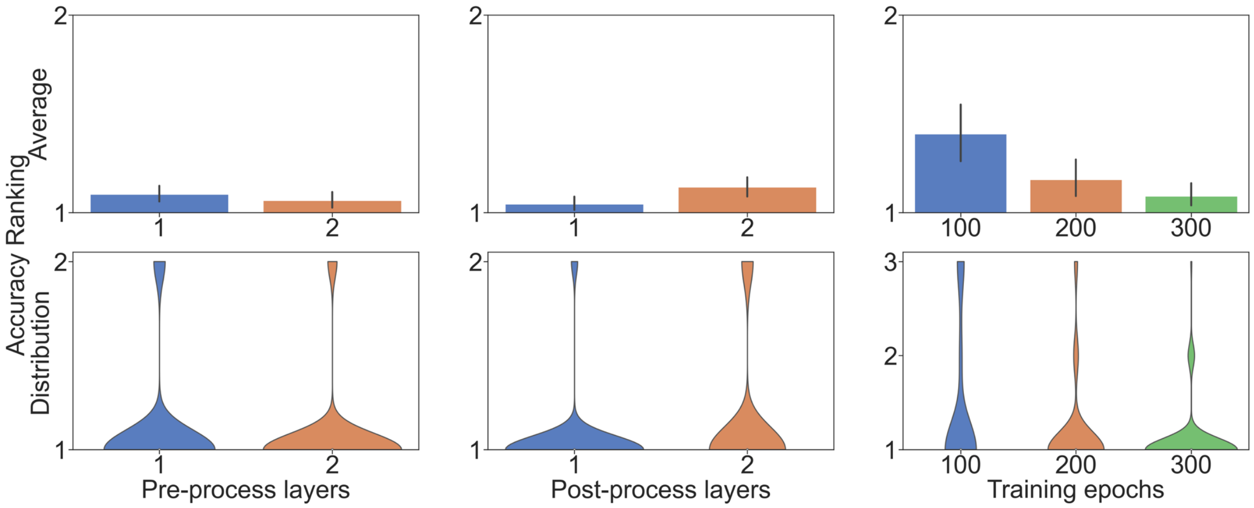

Figure S3 (a). ROC AUC Ranking analysis for Grape-Pi-GCNConv design in 6 dimensions. Lower is better. A tie was reached if designs had a ROC-AUC difference within 0.02.

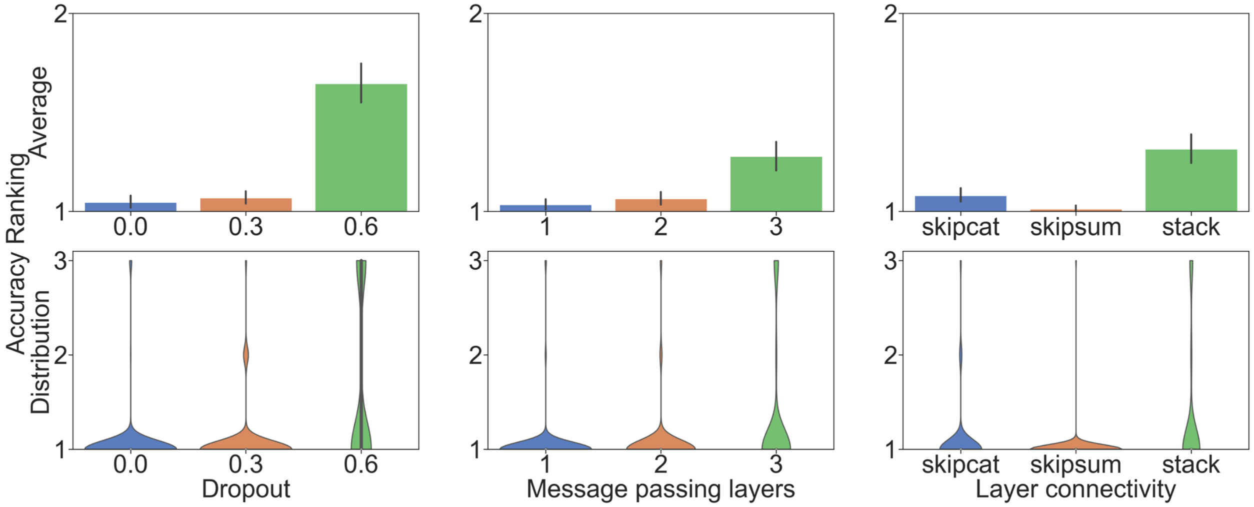

Figure S3 (b). ROC AUC Ranking analysis for Grape-Pi-SAGEConv design in 6 dimensions. Lower was better. A tie was reached if designs had a ROC-AUC difference within 0.02.

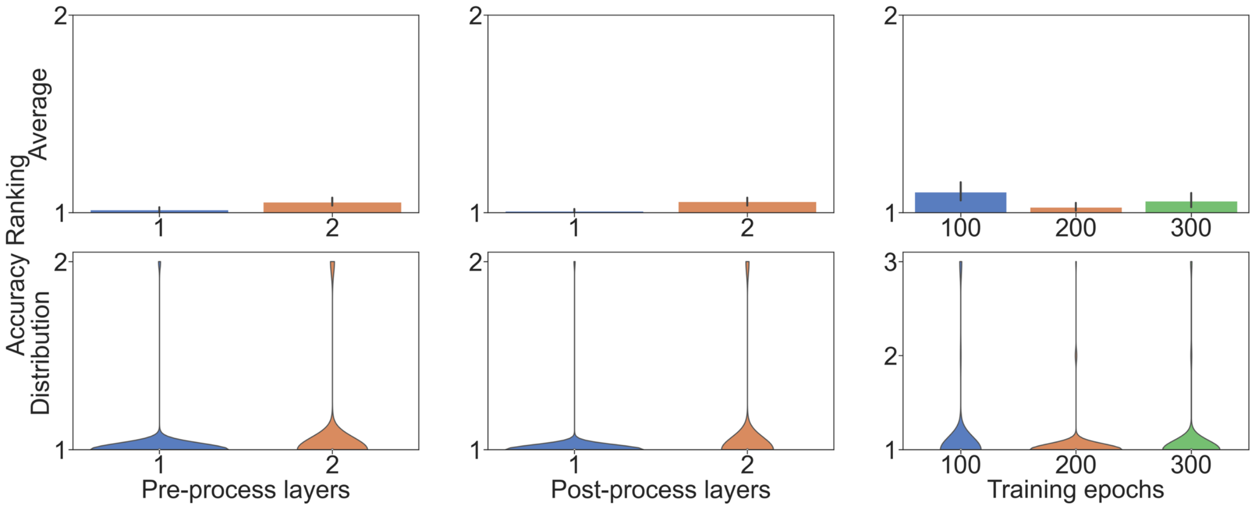

	Overall	Train set	Validation set	Test set	Unlabeled
Yeast-LCQ, Raw score as single feature	3,209	1,925	642	642	---
Yeast-OrbiTrap, Raw score as single feature	3,209	1,925	642	642	---
PCGC, Raw score as single feature	20,424	7523	2507	2507	7887

Table S2. Sample size in each dataset and train/validation/test splits. The sample size was defined as the number of (protein) nodes in a graph under the graph neural network setting. PCGC: primary cell gastric cancer

	ROC AUC		
Dataset	MLP (Benchmark)	GrapePi-GCNConv	GrapePi-SAGEConv
Yeast-LCQ	0.63	0.72	0.69
Yeast-LCQ with mRNA	0.82	0.86	0.86
Yeast-ORBI	0.84	0.88	0.88
Yeast-ORBI with mRNA	0.88	0.91	0.92
Gastric cancer Sample	0.82	0.91	0.91
Gastric cancer Sample with mRNA	0.91	0.92	0.94

Table S3: Performance of GrapePi-SAGEConv and GrapePi-GCNConv with the best hyperparameter combination on three tested datasets.

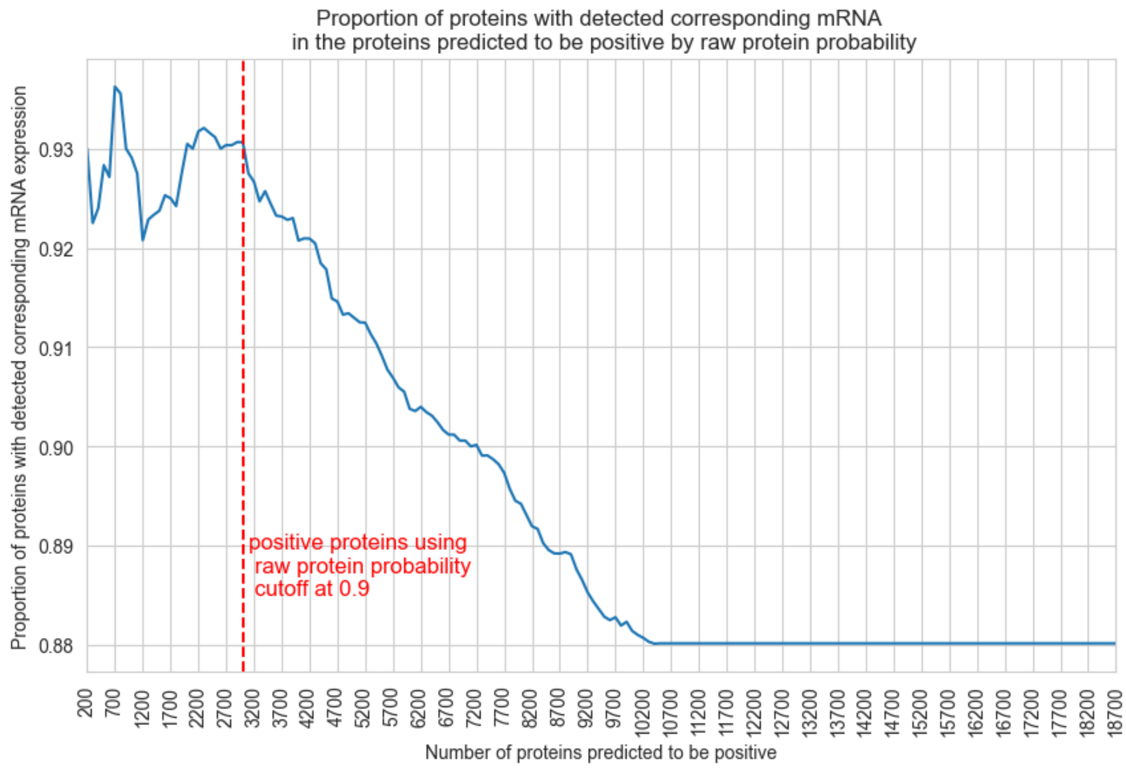

Figure S4. The proportion of proteins with detected corresponding mRNA for all proteins in the sample with different cutoffs of raw protein probability.

A

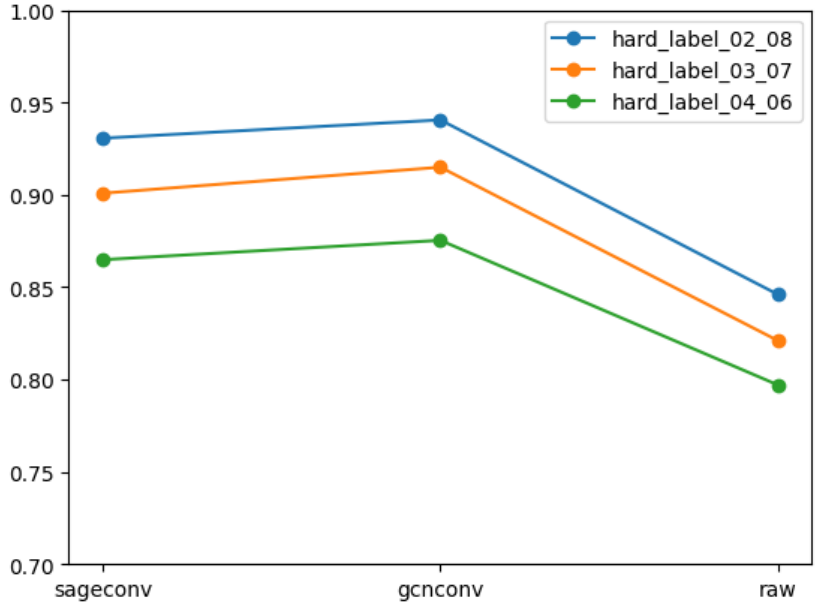

B

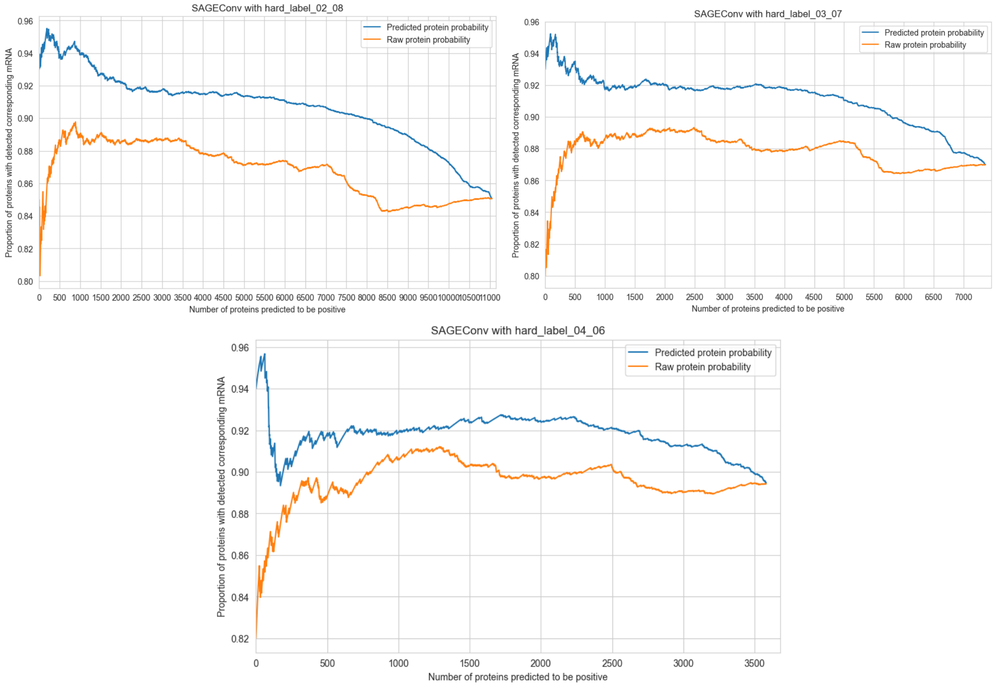

Figure S5. Sensitivity analysis for using different labeling criteria. Three different criteria for deciding positive and negative proteins have been tested: 1. Average protein probability <= 0.2 as negative; average protein probability >= 0.8 as positive; otherwise, unlabeled. 2. Average protein probability <=0.3 as negative; average protein probability >=0.7 as positive; otherwise, unlabeled; 3. Average protein probability <=0.4 as negative and average protein probability >=0.6 as positive; otherwise, unlabeled.

Graph neural network methods incorporating interaction information constantly outperform the raw protein probability by similar scales. A) AUC comparison between results in labeled test proteins: hard_label_02_08, hard_label_03_07, and hard_label_04_6 representing the ground-truth label created based on the three criteria above respectively. B) the mRNA coverage comparison for unlabeled unconfident proteins.

A

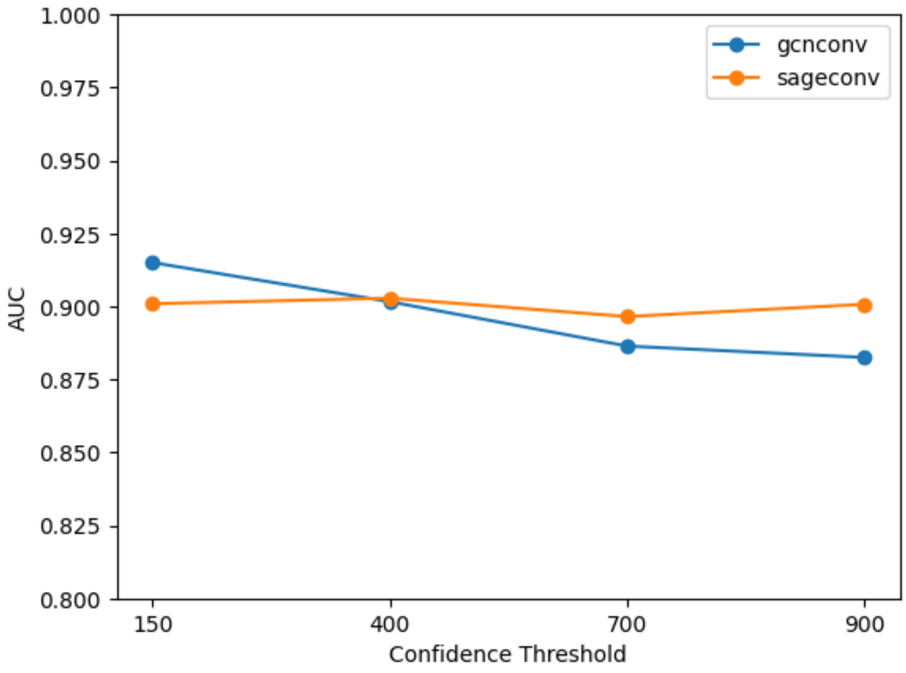

B

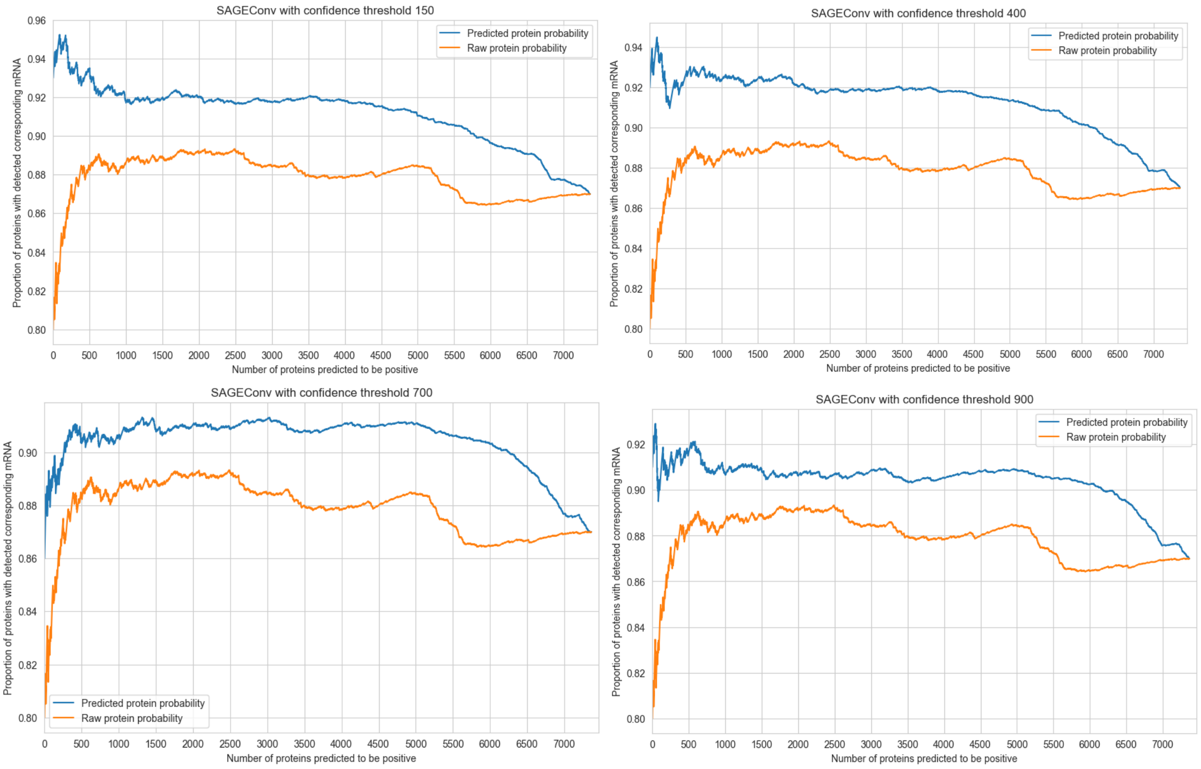

Figure S6. Sensitivity analysis for using different interaction confidence thresholds with data from the STRING database. Different thresholds on the STRING protein interaction dataset have been tested 1. Confidence score above 150 (low); 2. Confidence score above 400 (medium); 3. Confidence score above 700 (high) and confidence score above 900 (highest). A) AUC comparison between results in labeled test proteins; B) the mRNA coverage comparison for unlabeled unconfident proteins.

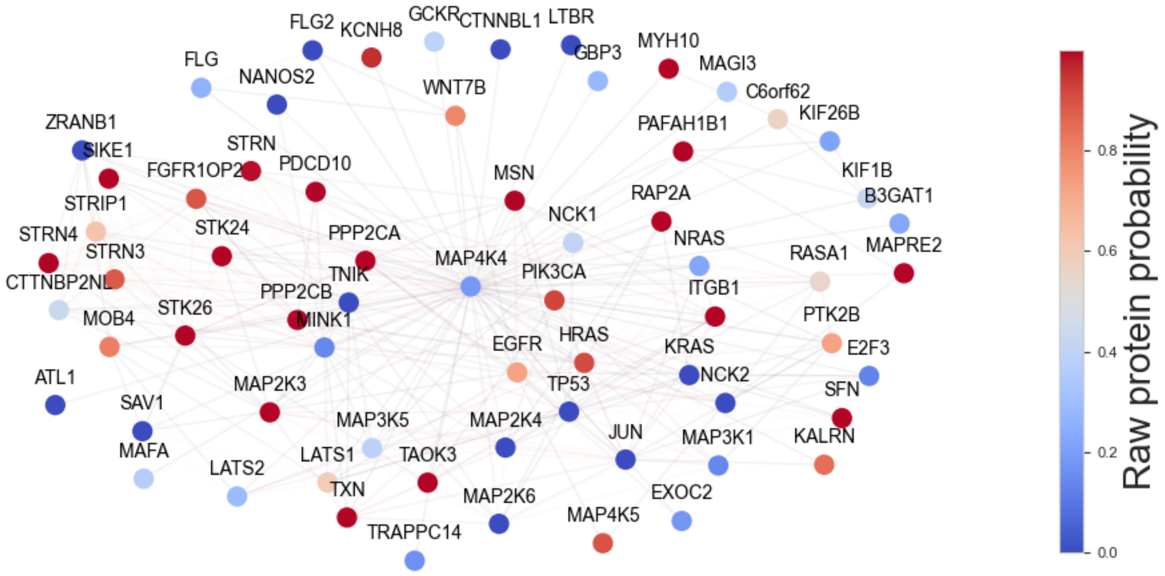

Figure S7. Illustration of model decision for promoted protein. Sub-network within one hop from the center protein O95819 (gene: MAP4K4). The exact figure with the gene symbol as a node label can be found in Figure S7.

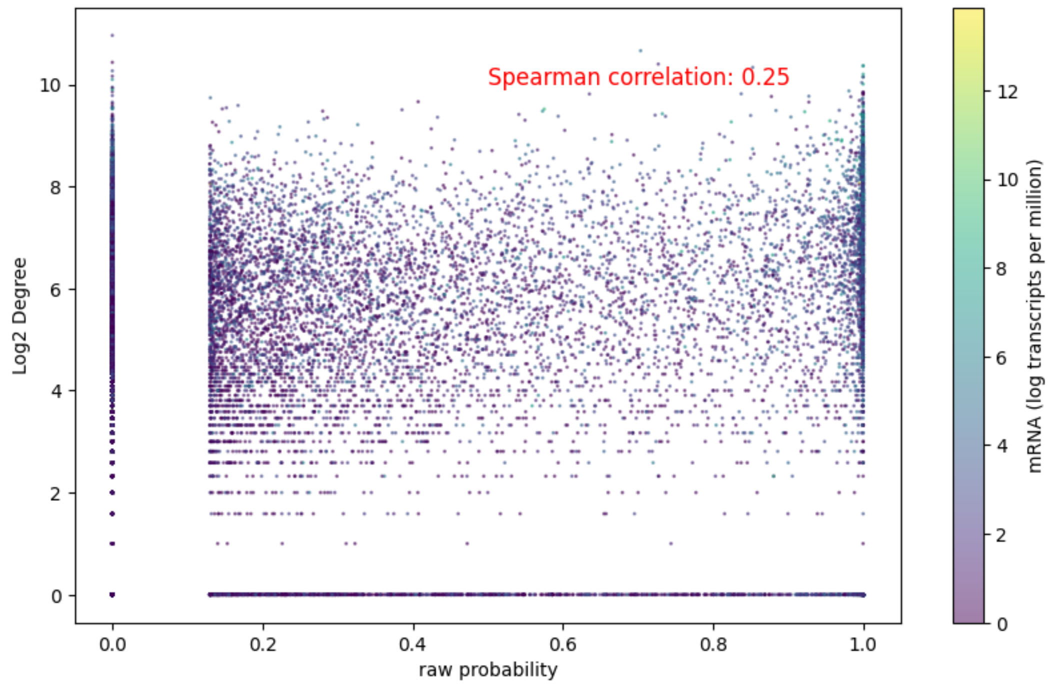

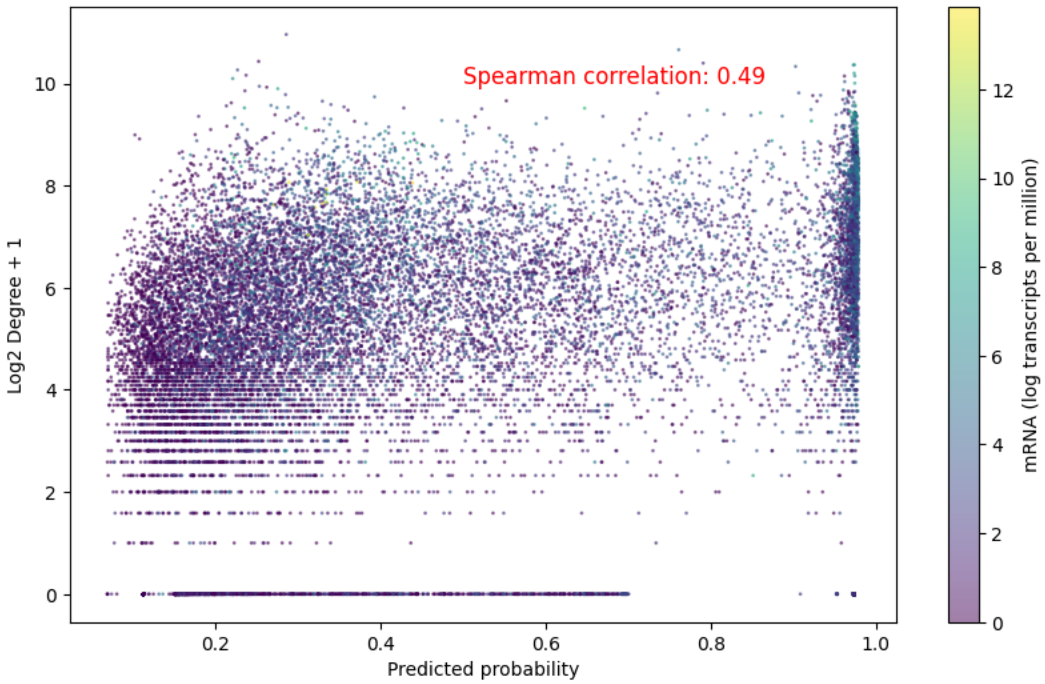

Figure S8. Correlation between node degree (number of interactors) and protein probability. Upper: the scatter plot and Spearman correlation between raw probability and node degree. Lower: the scatter plot and Spearman correlation between predicted probability and node degree.

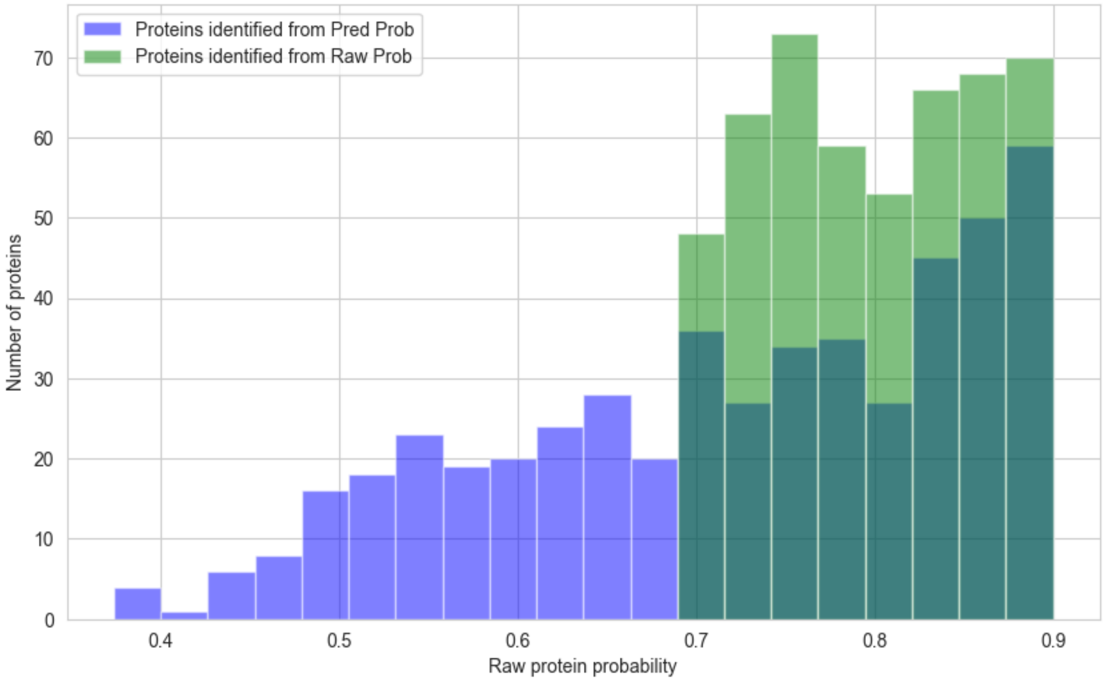

Figure S9. The distribution of original evidence for newly identified proteins based on predicted and raw probability. Newly identified proteins from predicted probability were across the original confidence, with fewer proteins from lower confidence.

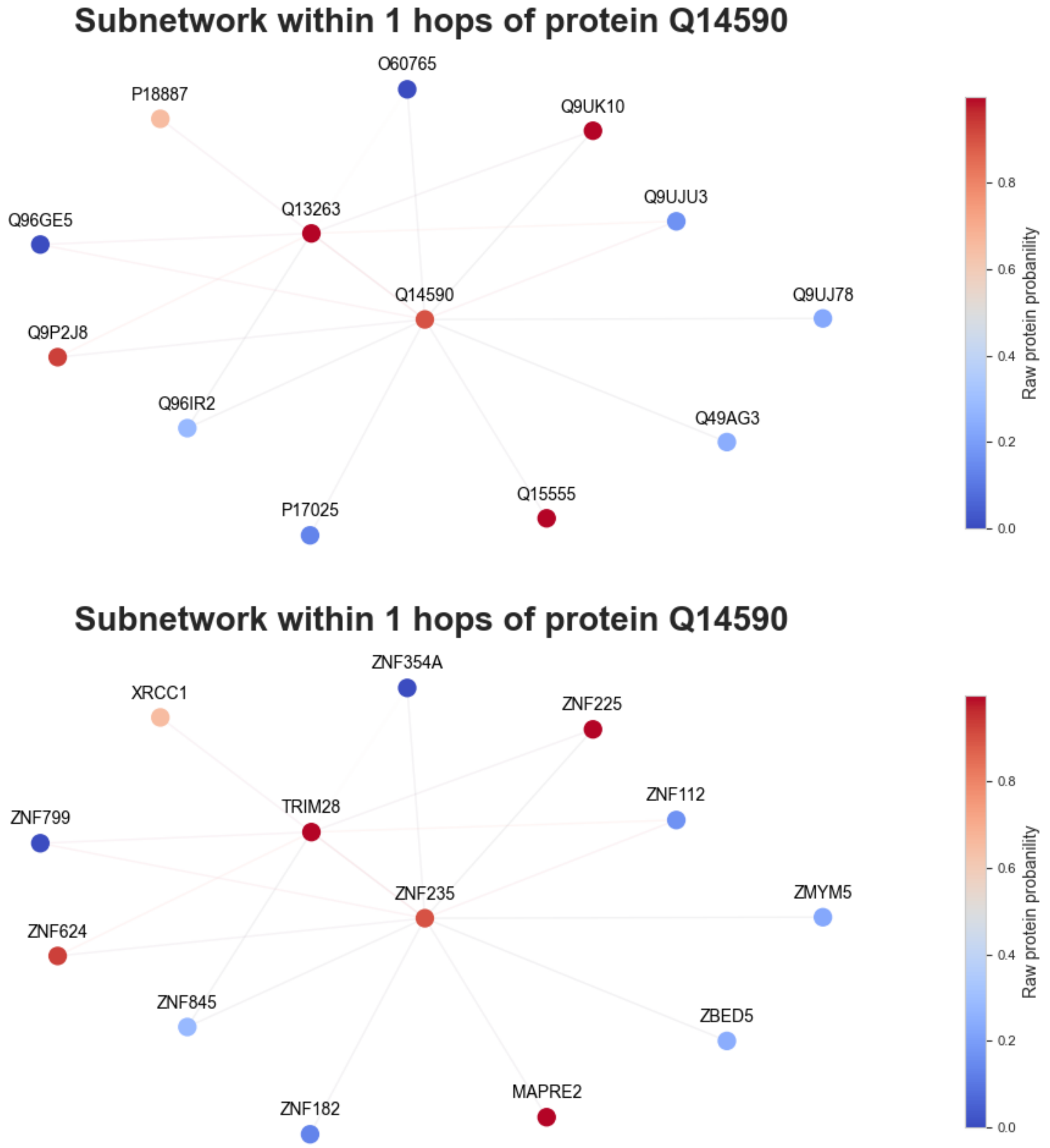

Figure S10. Illustration of model decision for a demoted protein. Sub-network within one hop from the center protein Q14590 (gene: ZNF235). The protein has a raw

probability of 0.90 and a predicted probability of 0.695. Upper: node label using UniProt accession. Lower: node label using gene symbol.

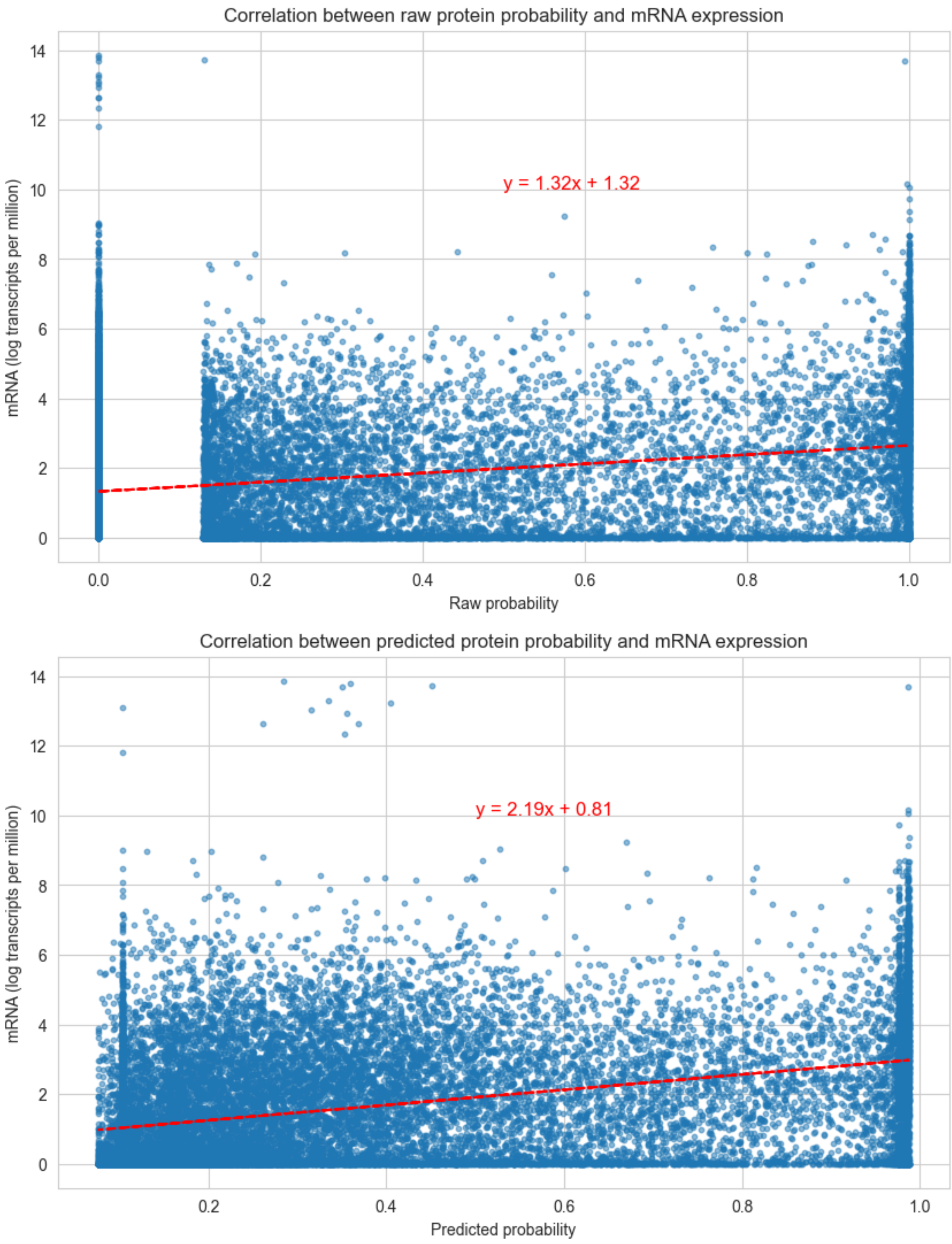

Figure S11. Correlation between protein probability and mRNA expression. Upper: correlation between raw probability and mRNA expression. Lower: correlation between

predicted probability and mRNA expression. It showed a stronger correlation between the predicted probability and mRNA expression than the correlation between raw probability and mRNA.

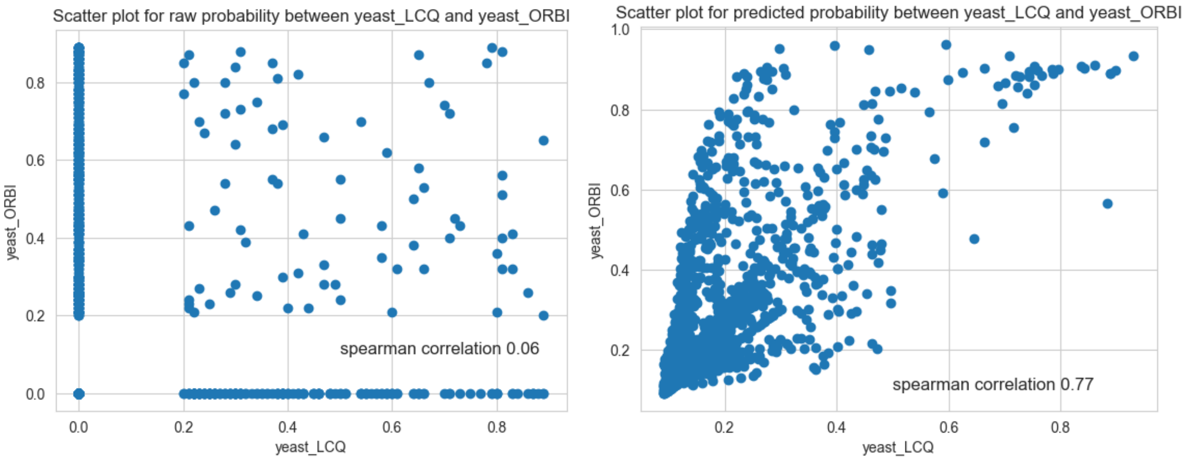

Figure S12. Consistency assessment for Grape-Pi predicted probability. The assessment was to check that the model prediction is consistent for datasets of similar conditions. A model was trained on the Yeast-rich-medium ORBI dataset using raw probability and mRNA, then the predicted probability for all proteins. The trained model was directly applied to the yeast-rich-medium LCQ dataset to get the predicted probability for all proteins. We check the correlation for proteins with raw probability < 0.9 in both datasets to check the consistency for their common non-confident proteins. Left: the correlation between their raw probabilities. Right: the correlation between their predicted probabilities. The predicted probabilities demonstrate a much better consistency over samples of similar conditions. A similar weaker pattern (0.41 vs 0.06) was observed when only raw probability was used as the input.

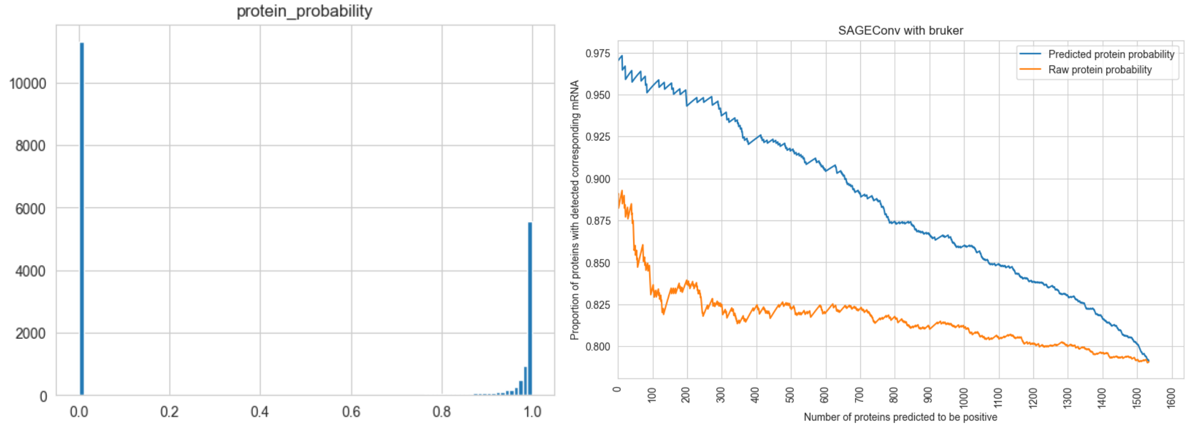

Figure S13. Validation of the model on Cell-line gastric cancer from Bruker MS. The model trained on the primary-cell gastric cancer (PCGC) dataset was applied to the cell-line data from the Bruker mass spectrometry instrument. Proteins with a raw probability of less than 0.9 but greater than 0.1 were considered as unconfident

proteins. For those unconfident proteins, we then ranked predicted probability and raw probability in ascending order. The proportion with corresponding mRNA expression (mRNA coverage rate) when gradually increasing the number of newly identified proteins. A consistently higher mRNA coverage rate indicates a better detection power.

Reference

Li, J., Zimmerman, L. J., Park, B. H., Tabb, D. L., Liebler, D. C., & Zhang, B. (2009). Network-assisted protein identification and data interpretation in shotgun proteomics. *Molecular Systems Biology*, *5*. https://doi.org/ARTN 303
10.1038/msb.2009.54
